# Discovery of a new family of relaxases in Firmicutes bacteria

**DOI:** 10.1371/journal.pgen.1006586

**Published:** 2017-02-16

**Authors:** Gayetri Ramachandran, Andrés Miguel-Arribas, David Abia, Praveen K. Singh, Isidro Crespo, César Gago-Córdoba, Jian An Hao, Juan Roman Luque-Ortega, Carlos Alfonso, Ling J. Wu, D. Roeland Boer, Wilfried J. J. Meijer

**Affiliations:** 1 Centro de Biología Molecular "Severo Ochoa" (CSIC-UAM), Instituto de Biología Molecular "Eladio Viñuela" (CSIC), Universidad Autónoma, Canto Blanco, Madrid, Spain; 2 XALOC beamline, ALBA synchrotron Light Source, Cerdanyola del Vallès, Barcelona, Spain; 3 Centro de Investigaciones Biológicas (CSIC), Madrid, Spain; 4 Centre for Bacterial Cell Biology, Institute for Cell and Molecular Biosciences, Newcastle University, Newcastle Upon Tyne, United Kingdom; Uppsala University, SWEDEN

## Abstract

Antibiotic resistance is a serious global problem. Antibiotic resistance genes (ARG), which are widespread in environmental bacteria, can be transferred to pathogenic bacteria via horizontal gene transfer (HGT). Gut microbiomes are especially apt for the emergence and dissemination of ARG. Conjugation is the HGT route that is predominantly responsible for the spread of ARG. Little is known about conjugative elements of Gram-positive bacteria, including those of the phylum Firmicutes, which are abundantly present in gut microbiomes. A critical step in the conjugation process is the relaxase-mediated site- and strand-specific nick in the *oriT* region of the conjugative element. This generates a single-stranded DNA molecule that is transferred from the donor to the recipient cell via a connecting channel. Here we identified and characterized the relaxosome components *oriT* and the relaxase of the conjugative plasmid pLS20 of the Firmicute *Bacillus subtilis*. We show that the relaxase gene, named *rel*_*LS20*_, is essential for conjugation, that it can function *in trans* and provide evidence that Tyr26 constitutes the active site residue. *In vivo* and *in vitro* analyses revealed that the *oriT* is located far upstream of the relaxase gene and that the nick site within *oriT* is located on the template strand of the conjugation genes. Surprisingly, the Rel_LS20_ shows very limited similarity to known relaxases. However, more than 800 genes to which no function had been attributed so far are predicted to encode proteins showing significant similarity to Rel_LS20_. Interestingly, these putative relaxases are encoded almost exclusively in Firmicutes bacteria. Thus, Rel_LS20_ constitutes the prototype of a new family of relaxases. The identification of this novel relaxase family will have an important impact in different aspects of future research in the field of HGT in Gram-positive bacteria in general, and specifically in the phylum of Firmicutes, and in gut microbiome research.

## Author summary

Antibiotics have saved the lives of millions. However, the emergence and spread of antibiotic resistance compromises the effectiveness of antibiotics. Genes conferring antibiotic resistance are often located on genetic elements that can be transferred to other bacteria. Conjugation is the predominant route responsible for spreading antibiotic resistance genes, and depends critically on a class of proteins called relaxases. These enzymes initiate conjugation by processing the DNA of the mobile element and are therefore an Achilles´ heel of the conjugation process. Although antibiotic resistance is an important health threat in both Gram-negative and Gram-positive bacteria, conjugation has so far been studied primarily in Gram-negative bacteria. Due to the extremely high concentration of bacteria, which favors conjugation, the intestinal gut is a hotspot for spreading antibiotic resistance. It is now known that a large part of the gut microbiome corresponds to Gram-positive bacteria, and many of these belong to the phylum Firmicutes. To better understand conjugation and specifically relaxases of Gram-positive conjugative elements we have identified and characterized the relaxase and the DNA region at which it acts of the conjugative plasmid pLS20 from the Gram-positive Firmicute *Bacillus subtilis*. We also show that the relaxase of pLS20 is the founding member of a new and large family of relaxases that is almost exclusively present in Firmicutes bacteria. This work will have important implications for the transfer of genes in Gram-positive bacteria in general and specifically for conjugation-mediated spread of antibiotic resistance in Firmicutes bacteria of the intestinal gut.

## Introduction

Horizontal gene transfer (HGT) processes are responsible for the spread of antibiotic resistance (Ab^R^) which poses a serious economic and health problem worldwide. Conjugation, which is the process by which a DNA element is actively transferred from a donor cell to a recipient cell through a dedicated transportation pore connecting the cells, is the main HGT route responsible for spreading Ab^R^ genes [1–4]. Conjugative elements can be embedded in a bacterial chromosome or they can be present on plasmids. Several aspects of conjugation have been studied in considerable depth over the last few decades. However, most conjugation studies concern Gram-negative (G-) bacteria. Recent studies provide evidence that the gut microbiome of humans and animals functions as a pool of Ab^R^ genes [5–9]. Bacteria from diverse environments and from food enter the gut, and the enormous numbers and density of microbes in the gut favors HGT, especially conjugation. The gut microbiome contains both G- and Gram-positive (G+) bacteria. A large fraction of the G+ bacteria of the gut microbiome belong to the phylum of Firmicutes [10]. Moreover, Firmicutes constitute a large fraction of the microbiota of fermented foods, many of which also thrive in the gut, and for which it has been shown that they can harbor Ab^R^ genes [11]. Together, this illustrates the need to better understand the biology of plasmid-mediated conjugation in G+ bacteria in general, and in the phylum of Firmicutes in particular.

We have been studying the low-copy number conjugative plasmid pLS20, originally isolated from the Firmicutes bacterium *B*. *subtilis natto* strain IFO3335 that is used in the fermentation of soybeans to produce “natto”, a popular dish in South Asia [12]. pLS20 is known to be conjugative in liquid media as well as on solid media [13–15]. The replication region of pLS20 has been determined [16] and is flanked at its right side by three genes that are involved in regulating the expression of the large conjugation operon that is located immediately downstream of these three genes [17,18].

The basis of the conjugation process is conserved in plasmids of G+ and G- bacteria [19–21]. Thus, in most cases, only one DNA strand, called the T-strand, is transferred into the recipient cell through a sophisticated, multi-component pore, -a type IV secretion system (T4SS)-, that connects the donor and recipient cells. Initiation of the generation of the T-strand and its delivery to the pore involves a specific nucleoprotein complex, called the relaxosome. The pivotal component of the relaxosome is a relaxase that recognizes and binds to specific sequences within a region of several hundred base pairs on the plasmid named the origin of transfer *(oriT)*. Often, the *oriT* region is also recognized by auxiliary proteins, which are generally encoded by the plasmid but can also involve host-encoded proteins [for review see, 21]. The relaxase cleaves the DNA in a strand- and site-specific manner at a specific position called the *nic* site within the *oriT* region. The generated hydroxyl group at 3´-end of the nick site functions as a primer for DNA elongation. Upon nicking, the relaxase remains covalently attached to the 5´-end of the nicked T-strand via a tyrosine residue, and this complex is delivered to the so called T4 Coupling Protein (T4CP) that is located at the cytoplasmic side of the transferosome and which is actively involved in the transfer of the relaxase and the attached T-strand into the recipient cell. Besides conjugative plasmids, there is a group of plasmids that lack the T4SS but contain a relaxase gene and an *oriT*, and sometimes auxiliary relaxasome genes. These plasmids, called mobilizable plasmids, can be transferred to recipient cells when they are co-resident with a conjugative plasmid that provides the T4SS.

The essential role of relaxases in the transfer of plasmids and other conjugative elements gain them much attention and several relaxases have been studied at the functional, biochemical and structural level. In addition, they have been chosen as a target to develop drugs interfering with its activity [22]. Based on their relationship, relaxases have been classified into six families [23,24]. However, as for other aspects of conjugation, most of our knowledge on conjugative relaxases is based on those that are encoded by mobilizable or conjugative plasmids of G- origin, or those on mobilizable plasmids of G+ origin [23,24].

Here we describe the identification of relaxosome components of the *B*. *subtilis* plasmid pLS20. Contrary to many other plasmids, the relaxosome components of pLS20 are located within its large conjugation operon. We identified the relaxase gene of pLS20cat, which we name *rel*_*LS20*_, and showed that it is essential for conjugation. We also defined the minimal functional *oriT* region of pLS20cat, *oriT*_*LS20*_, and demonstrate that it contains a pronounced static bent. We then demonstrated that Rel_LS20_ has nicking-activity and determined the nick site in *oriT*_*LS20*_. Interestingly, Rel_LS20_ shows only very limited homology with known relaxases and cannot be classified into one of the six existing relaxase families. Importantly, we found that more than 800 deduced proteins present in public databases for which no function had been attributed, and which are almost all encoded by bacteria belonging to the phylum of Firmicutes, show significant homology with Rel_LS20_. Most probably, many or all of these proteins are relaxases, and hence Rel_LS20_ constitutes the prototype of a new family of relaxases that we name MOB_L_. Thus, besides important progress in the understanding of the relaxosome components of pLS20, our studies have important implications for a large number of related relaxases within the phylum Firmicutes.

## Results

### Identification of putative relaxase gene of pLS20cat by *in silico* analysis

Putative function(s) of newly sequenced genes are commonly assigned based upon similarity of the entire or partial region(s) of the deduced protein sequence to proteins, protein domains or signatures present in the conserved domain database (CDD) of the NCBI [25]. Surprisingly, none of the deduced pLS20cat proteins was identified as being a putative relaxase after searching pLS20cat against the CDD. We therefore screened all the putatively encoded proteins of pLS20cat for the presence of one or more motifs that are distinctive of relaxases previously described and classified into six MOB families, MOB_P_, MOB_Q_, MOB_F_, MOB_C_, MOB_H_ and MOB_V_ [23,24], considering that these motifs could include sequences that are not covered by the twelve relaxase-specific motifs present in the CDD. Generally, these motifs concern regions that have a crucial role in the enzymatic activity of the protein. For instance, motif I and III correspond respectively to regions that contain the catalytically active tyrosine(s), and the three histidines essential for coordinating a divalent metal ion [24]. This screening led to the identification of one candidate, gene *58*, as a potential relaxase gene. Interestingly, unlike many other conjugative plasmids, the pLS20 putative relaxase gene is not located adjacent to the gene predicted to encode T4CPs [24], pLS20cat gene *48*.

**Fig 1:**
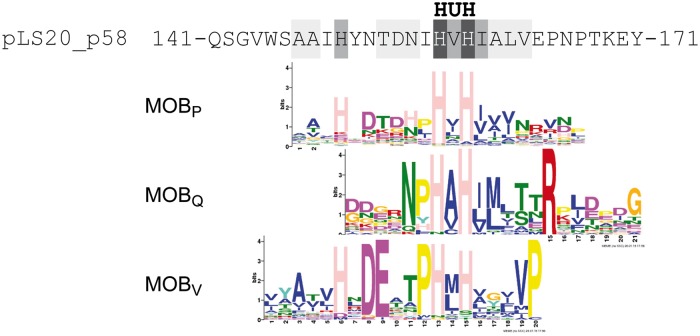
A 17 residue region of the deduced protein sequence of pLS20cat gene *58* shows similarity to the motif III regions of MOB_P_, MOB_Q_ and MOB_V_ type relaxases. pLS20cat has been sequenced independently in our lab and in the lab of M. Itaya (Keio University, Japan) who deposited the sequence in public databases where it was given the accession number NC_015148.1. pLS20cat protein p58, according to our nomenclature, has been assigned the accession number YP_004243525.1. Residues 141–171 of the deduced pLS20cat protein p58 are aligned with the consensus sequences, represented as Weblogos, of the motif III region of relaxases belonging to the MOB_P_, MOB_Q_ and MOB_V_ family. The consensus sequence of the motif III regions of the published relaxases of each MOB family [24] was identified here with the motif-identification program MEME [27]. The position of the HUH signature is given at the top. Residues of the deduced pLS20cat p58 sequence are highlighted against a light grey, dark grey and black background when they are conserved with respect to the consensus sequence present in one, two or three of the MOB families, respectively.

Although the overall similarity is limited, a stretch of 17 amino acids (residues 147–163) of the deduced protein p58 sequence showed similarity to part of motifs III present in many relaxases. Relaxase motif III embraces the “HUH” sequence that is characteristic of the HUH endonuclease superfamily, which includes many relaxases [26]. The “HUH” sequence is essential for enzymatic activity of the protein. Within the HUH motif the “U” represents a bulky hydrophobic residue, and “H” a Histidine. The two His residues in this motif correspond to two of the three ligands that coordinate a divalent metal ion that is required for the enzymatic nicking-closing activity. In the case of relaxases, the third ligand is often also a Histidine. This third His residue is located near the HUH motif in the primary sequence and is included in the motif III of relaxases, which therefore is also referred to as the 3H motif. Thus, except those belonging to the MOB_H_ or MOB_C_ family, all other relaxases contain a motif III [24]. Fig 1 shows the similarity between residues 141–171 of the deduced pLS20cat protein p58 and the consensus sequences of motif III regions present in relaxases belonging to the MOB_P_, MOB_Q_ and MOB_V_ families. Interestingly, no significant similarity exists between p58 and the consensus sequence of motif III of the MOB_F_ type relaxases (see Discussion). In summary, *in silico* analyses suggested that pLS0cat gene *58* may encode a relaxase.

### pLS20cat gene *58* is the prototype of a new family of genes whose orthologs are widespread in Firmicutes bacteria

If pLS20cat gene *58* indeed encodes for a relaxase then it could be the founding member of a novel relaxase family. To test this possibility we performed a psi-blastp search of the NCBI nr database using the deduced protein p58 sequence as a query (see Materials and methods for details). After removing redundant sequences this search resulted in the identification of 817 hits showing significant similarity with putative pLS20cat protein p58 (threshold E-value *1e-15*, see S1 Table for a list of these hits). Interestingly, almost all of the identified sequences corresponded to products of (putative) genes of bacteria belonging to the phylum Firmicutes (99.6% Firmicutes, 0.1% bacteriodetes, 0.1% tenericutes and 0.1% undefined). The vast majority (99.6%) of these hits were designated as hypothetical protein. However, three hits were labelled as (putative) relaxase: WP_014386599.1 (putative relaxase of *Lactococcus garvieae* 21881 plasmid pGL5 [28]), WP_008381272.1 (putative relaxase of *Enterococcus* sp. C1 [29]), and WP_011377372.1 (putative relaxase of *Enterococcus faecium* plasmid pHTβ [30]). In the latter case, genetic evidence suggests that the corresponding gene may encode the relaxase of plasmid pHTβ. Firstly, these observations lend further support to the view that pLS20cat gene *58* encodes a relaxase. Based on this and on results described below we name gene *58 rel*_*LS20*_. Secondly, these observations provide evidence that Rel_LS20_ is the prototype of a large, new family of relaxases, which we name MOB_L_, and which contains over 800 members that are almost exclusively encoded by Firmicutes bacteria.

We next performed additional analyses to gain insights into the evolutionary relationship between the newly identified MOB_L_ family of relaxases and the previously described relaxase families. The motif-identification program MEME [27] was used to identify up to 10 conserved motifs in sequences of the MOB_L_ members (E-value <1e-15, for details see Materials and methods). To discriminate the motifs identified in our work from those described previously for the six MOB families [24], we refer to the MEME motifs determined in this work as signatures. Weblogo presentations of the ten identified signatures of the MOB_L_ type relaxases are shown in S1A Fig. The positions of the signatures in the Rel_LS20_ primary sequence are given in S1B Fig.

Similarly, we analysed the relaxases of the six previously defined MOB families and identified up to ten signatures in each family (see S2 Fig). We then used the Motif Alignment and Search Tool (MAST) of the MEME suite to determine the presence of (i) MOB_L_ signatures in the relaxases of the other six MOB families (S2 Table), and (ii) signatures of those six MOB families in members of the MOB_L_ family (S3 Table). None of the MOB_L_ signatures were detected in members of the MOB_C_ and MOB_H_ families. As explained above, MOB_L_ signature 1 -containing the conserved histidines-, shares similarity with motif III regions of MOB_Q_, MOB_V_ and MOB_P_ type relaxases (see Fig 1). Not surprisingly therefore, the MOB_L_ signature 1 was detected in a substantial number of relaxases of these MOB families but not in the members of the other three families. Importantly, other MOB_L_ signatures were either not detected or detected in only a limited number of relaxases from the six existing MOB families (see S2 Table). A similar tendency was observed in the reciprocal analyses (see S3 Table). Cross detection of signatures was most frequently observed between the MOB_L_ and MOB_P_ families. Altogether, these data corroborate the view that Rel_LS20_ and the identified related (putative) proteins constitute a new MOB family, and that MOB_L_ is closest related to the MOB_P_ family of relaxases.

### Members of the MOB_L_ family can be divided into two clades

Rel_LS20_ contains eight out of the ten MOB_L_ signatures (S1B Fig). Most other MOB_L_ members also contain most but not all MOB_L_ signatures, indicating that the MOB_L_ family is composed of different clades. To test this statistically we calculated a phylogenetic tree using neighbor-joining analysis, applying bootstrap values of 1000 replicates (see Materials and methods, S3 Fig). The phylogenetic tree obtained shows that the MOB_L_ members can be divided into two clades, and that Rel_LS20_ belongs to clade 1.

Bioinformatic analyses were performed to study whether, besides Rel_LS20_, other MOB_L_ members are encoded by a plasmid. For this we identified all circular plasmids deposited in public databases for which the complete sequence has been determined (see Materials and methods). This resulted in a database of 10,904 plasmids. Next, the sequences of the 817 MOB_L_ relaxases were used as query to search the translated sequences of the generated plasmid database for similarity with stringent settings (>95% similarity over >80% of the entire protein sequence). This analysis indicated that at least 306 of the 817 MOB_L_ members are located on a plasmid. Interestingly, 98% of identified plasmid-located *mob*_*L*_ genes (300 genes) correspond to members classified in clade 1. Most of the DNA sequences currently present in databases was generated from metagenomic or by shotgun sequencing approaches, which do not discriminate between chromosomal or extrachromosomal DNA. It is therefore plausible that (many) more clade 1 MOB_L_ genes are located on plasmids. To gain insight into the nature of the relaxases belonging to MOB_L_ clade 2, we performed *in silico* analysis and manually inspected DNA regions surrounding these genes. However, we were not able to draw firm conclusions about the nature of the clade 2 MOB_L_ members, largely because very little functional information is available about these genes.

In summary, members of the newly identified MOB_L_ family can be subdivided into two clades and many members belonging to clade 1 are located on plasmids. Strikingly, all these genes are almost exclusively present in the phylum Firmicutes.

### pLS20cat gene *58* is essential for conjugation

Since processing of DNA to produce the T-DNA strand that is transferred into the recipient cell is a vital step in the conjugation process, it was expected that gene *58* would be essential for conjugation if it encoded the relaxase of pLS20cat. Gene *58* is translationally coupled to the preceding gene *57*, which in turn is closely linked to gene *56* [17]. Therefore, we constructed a deletion derivative of pLS20cat lacking these three genes (see Materials and methods) and named the resulting plasmid pLS20Δ56–58. Next, *B*. *subtilis* 168 containing pLS20cat (strain PKS11) or pLS20Δ56–58 (strain GR149) were used as donor cells to determine their conjugation efficiencies using a standard protocol (see Materials and methods). The efficiency of conjugation observed for pLS20cat was in the range of 10^−3^, which is similar to values reported previously under these conditions [17,18]. However, no transconjugants were observed for pLS20Δ56–58 (see Table 1). These results suggested that at least one of the genes of *56* to *58* is required for conjugation. However, it was also possible that the deleted region contained the *oriT*, which is essential for conjugation. To rule out this possibility, we re-introduced genes *56*–*58*, under the control of an IPTG-inducible P_*spank*_ promoter, into strain GR149 (harbouring pLS20Δ56–58) at the *amyE* locus. As expected, no transconjugants were obtained when the resulting strain, GR150, was grown in the absence of IPTG. Importantly, conjugation efficiencies similar to those obtained with pLS20cat were obtained for pLS20Δ56–58 when GR150 was grown in the presence of IPTG (see Table 1). These results demonstrated therefore that one or more of the pLS20cat genes *56*, *57* and *58* were required for conjugation. In addition, the results showed that (i) pLS20Δ56–58 contains a functional *oriT* that hence must be located outside the deleted DNA fragment spanning genes *56–58*, and (ii) that genes *56–58* can function in *trans*.

**Table 1:** Conjugation efficiencies of pLS20cat and pLS20Δ56–58 in different backgrounds.

Strain	Genotype	Plasmid	Inductor	Conjugation efficiency *
PKS11	168	pLS20cat	-	8.3x10^-3^
GR149	168	pLS20Δ56–58	-	< 10^−7^
GR150	168, *amyE*::P_*spank*_ *56–58*	pLS20Δ56–58	-	< 10^−7^
			+	1.4x10^-3^
GR206	168, *amyE*::P_*spank*_ *56–57*	pLS20Δ56–58	-	< 10^−7^
			+	< 10^−7^

*: Conjugation efficiencies are calculated as transconjugants/donor, and correspond to the mean value of at least three independent experiments. When indicated, the inductor IPTG was added at a final concentration of 1 mM.

To determine whether gene *58* is required for conjugation we constructed strain GR206 that contains genes *56* and *57* (but not *58*) under the control of the P_*spank*_ promoter at the *amyE* locus and harbouring pLS20Δ56–58. No transconjugants were obtained when this strain was used as donor in conjugation experiments, regardless of whether cells were grown in the presence or absence of IPTG. These results showed that pLS20cat gene *58* was required for conjugation, as would be expected for a relaxase gene.

### The origin of transfer region of pLS20cat, *oriT*_LS20_, is located upstream of gene *56* and is intrinsically bent

**Fig 2:**
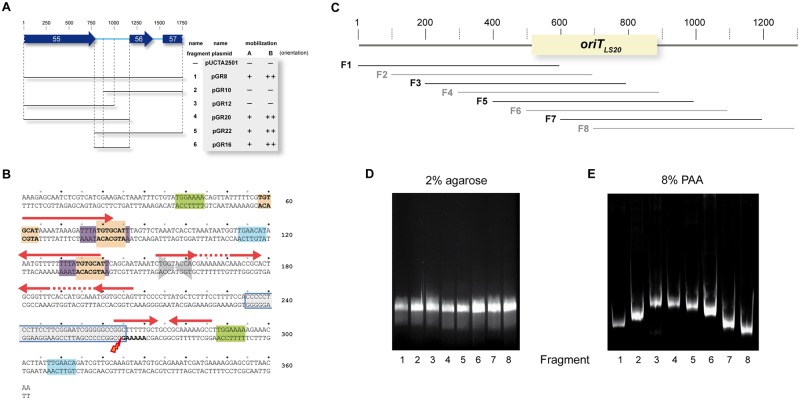
Determination and characteristics of *oriT*_*LS20*_. (A) Schematic overview of pLS20cat regions analyzed for the presence of a functional *oriT* region. The corresponding pLS20cat region and position of genes *55*, *56* and *57* are presented on the top. Numbers correspond to length in base pairs. The regions cloned are indicated by horizontal bars. The names of the fragments and the plasmids, and their ability to be mobilized are indicated at the right. “A” and “B” correspond to the orientation of the cloned fragment. -, + and ++: mobilization frequencies <10^−7^, in the order of 10^−5^, and 10^−4^, respectively. (B) Features of the *oriT*_*LS20*_ region. The sequence of the 362 bp *oriT*_*LS20*_ region is presented along with identified features. Inverted repeated sequences are indicated with red arrows. Identical or nearly identical direct repeated sequences are boxed in green, orange or purple. Note that the sequence with consensus 5´-TGTGCAT´-3 is present three times. The palindromic sequence 5´-TGGTACCA-3´ is indicated with two converging arrowheads. The 30-bp region with a GC-content of 73% is highlighted with a blue-lined box. Numbering of the fragment is given on the right. The determined *nic* site is indicated with a lightning symbol. (C) Schematic representation of the 1,300 bp pLS20cat region containing *oriT*_*LS20*_ (blue box) studied for the presence of a static bent. The lower part indicates the positions of the 600 bp DNA fragments F1 to F8 with respect to *oriT*_*LS20*_, and which were subjected to electrophoresis in gels of 2% agarose (D) or 8% native PAA (E). After electrophoresis, the agarose and PAA gels were stained with ethidium bromide.

The results presented above show that Rel_LS20_ can function in *trans*. Consequently, it was expected that pLS20cat could mobilize a compatible plasmid containing the *oriT* region of pLS20cat but lacking the relaxase gene. We used this strategy to identify the *oriT* region of pLS20cat, which we name *oriT*_LS20_ henceforth. Thus, we engineered the *oriT* screening vector pUCTA2501 and screened derivatives containing regions of pLS20 for their ability to be mobilized by pLS20cat. As expected, the empty vector pUCTA2501 was not mobilized by pLS20cat (see Table 2). The *oriT* regions are often located upstream of the relaxase gene [21,23], and the results presented above showed that pLS20Δ56–58 contained a functional *oriT*. We therefore first tested a 1.75 kb fragment, named Fragment 1, which encompasses the 3´-region of pLS20cat gene *55* till the middle of gene *57* (see Fig 2A for a schematic representation). For this, Fragment 1 was cloned in pUCTA2501 resulting in plasmids pGR8A and pGR8B (“A” and “B” correspond to the orientation of the cloned fragment tested). As shown in Table 2 and schematically in Fig 2A, pLS20cat was able to mobilize both plasmids pGR8A and B, indicating that *oriT*_LS20_ is located on the 1.75 kb Fragment 1. In addition, these results indicated that Rel_LS20_ can act on *oriT*_*LS20*_ regardless of its orientation in the plasmid. To delineate the *oriT*_LS20_ region further several internal regions of Fragment 1 were sub-cloned into pUCTA2501 and the resulting derivatives were tested for their ability to be mobilized by pLS20cat. Using this approach the *oriT*_*LS20*_ was delineated to a region of 362 bp (Fragment 6 present in vectors pGR16A/B) that corresponds to the intergenic region between genes *55*–*56* (see Table 2 and Fig 2A).

**Table 2:** Mobilization efficiencies of *oriT*-screening vector pUCTA2501 and derivatives.

Strain	Plasmid ^§^	Region cloned (bp)	Mobilization efficiency *
GR104	pUCTA2501		< 10^−7^
GR124	pGR8A	1739	3.3X10^-5^
GR183	pGR8B	1739	1.2X10^-4^
GR114	pGR10A	849	-< 10^−7^
GR121	pGR10B	849	< 10^−7^
GR115	pGR12A	949	< 10^−7^
GR122	pGR12B	949	< 10^−7^
GR184	pGR20A	1152	4.5X10^-5^
GR140	pGR20B	1152	1.7X10^-4^
GR139	pGR22A	949	6.8X10^-5^
GR185	pGR22B	949	1.2X10^-4^
GR137	pGR16A	362	2.0X10^-5^
GR138	pGR16B	362	1.5X10^-4^

^§^: Besides the plasmid mentioned, all strains contained pLS20cat.

*: Mobilization efficiencies are calculated as Em-resistant transconjugants/donor using strain PS110 as recipient strain. Mobilization efficiencies are the mean value of at least three independent experiments. The “A” and “B” extensions of the pUCTA2501 derivatives reflect different orientations of the same insert.

The topology of DNA, including intrinsic bents, can have major effects on the function of proteins that bind or process DNA [31,32]. The *oriT* regions often contain repeated sequences, and several *oriT* regions from conjugative elements of Gram-negative bacteria have been demonstrated to contain an intrinsic bent which is believed to be important for optimal binding and functionality of the relaxosome proteins [for review see, 21]. Fig 2B shows that *oriT*_*LS20*_ also contains several direct and inverted repeated sequences. *In silico* analysis of the entire pLS20cat sequence predicted the presence of a static bent near *oriT*_*LS20*_ (S4 Fig). To test whether *oriT*_*LS20*_ is intrinsically bent we performed circular permutation assays. Thus, eight DNA fragments, - F1-F8, each 600 bp long and corresponding to different positions of the *oriT*_*LS20*_ (see Fig 2C) -, were generated and their migration in 2% agarose or 8% native PAA gels was tested. As expected, identical migration positions were observed when the fragments were run in agarose gel (Fig 2D). However, when run on the native PAA gel clear differences in migration were observed between the fragments, demonstrating that the *oriT*_*LS20*_ region is intrinsically bent (Fig 2E). Taking into account that migration is most affected when the bent is located in the middle of a DNA fragment [33], these results suggest that the bent is located towards the 5´ region of *oriT*_*LS20*_.

### Rel_LS20_ forms monomers in solution

**Fig 3:**
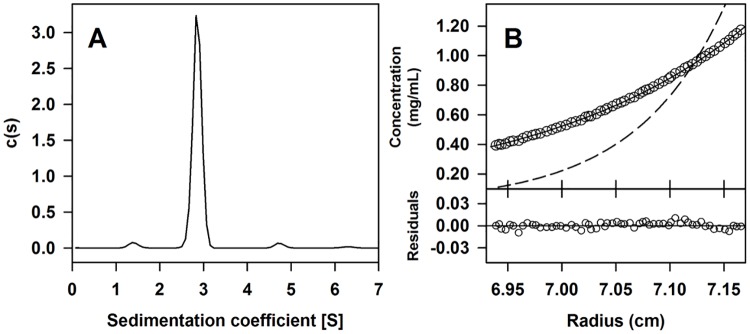
Analytical ultracentrifugation analyses showed that Rel_LS20_ is monomeric in solution. Purified Rel_LS20_ (12 μM) was studied by sedimentation velocity (SV) and sedimentation equilibrium (SE). Plot (A) represents the sedimentation coefficient distribution c(s) profile obtained by SV data analysis. Graph (B) shows the experimental data obtained by SE assays (empty circles) and best-fit analysis considering a protein monomer (black line) and dimer (dashed line) species model. Lower part represents the difference between experimental data and the best fit to a single species model (residuals).

To characterize the relaxase of pLS20cat *in vitro*, we purified Rel_LS20_ (Mw 50,329 Da) fused to a His_(6)_ tag at its C-terminus from *E*. *coli*. We first determined its oligomerization state using two complementary analytical ultracentrifugation approaches, i.e. sedimentation velocity (SV) and sedimentation equilibrium (SE) (Fig 3), as well as dynamic light scattering (DLS) experiments using the same experimental conditions.

More than 95% of the Rel_LS20_ absorbance in SV experiments corresponded to a narrow peak with an experimental sedimentation coefficient of 2.8 S (*s*_20,*w*_ = 3.3 S), matching the theoretical behaviour of the moderately elongated protein monomer (f/f_0_ = 1.5) (Fig 3A). DLS analysis gave a D value of 56.7 ± 0.8 μm^2^/s. Applying the obtained D and S values for Rel_LS20_ to the Svedberg equation resulted in an apparent molecular mass of 47,760 Da, which is close to the theoretical monomer molecular mass (from amino acid composition). In SE experiments a calculated average molecular mass of 43,200 ± 300 Da was obtained, a value slightly lower than the monomer molecular mass. The experimental data adjust very well to the best-fit line assuming Rel_LS20_ being a monomer (Fig 3B). In summary, the outcome of three complementary experimental approaches showed that Rel_LS20_ is monomeric in solution.

### Biochemical analyses of Rel_LS20_ and determination of the *nic* site

**Fig 4:**
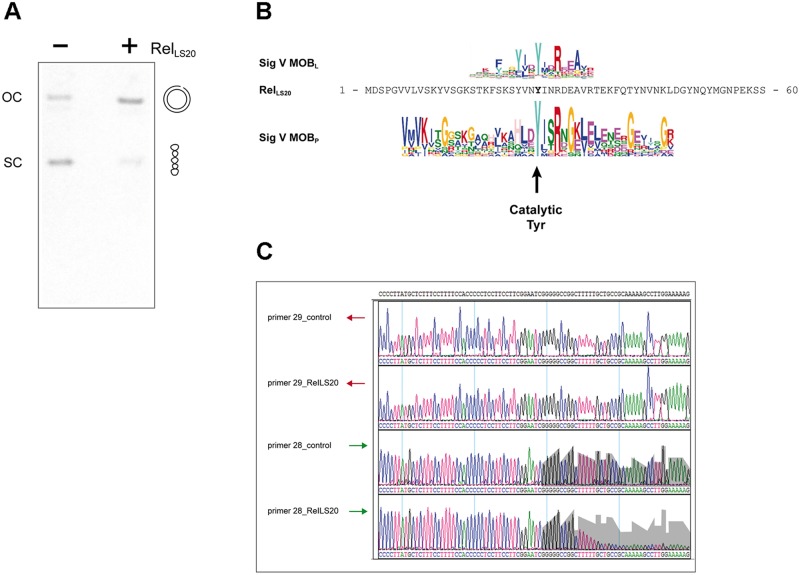
Determination of the catalytically active tyrosine residue of Rel_LS20_, and the position of the nick site within oriT_LS20_. (A) Plasmid pGR16B containing *oriT*_*LS20*_ was incubated in a buffer lacking (-) or containing (+) purified Rel_LS20_. Next, both samples were treated with proteinase K and DNA was run on a 0.8% agarose gel. The positions of supercoiled (sc) and nicked open circular DNA (oc) are indicated. (B) The N-terminal 60 residues of Rel_LS20_ are aligned with the consensus sequences (presented as weblogos) of signatures 5 of the MOB_L_ and MOB_P_ type relaxases. The position of the catalytic Tyrosine residue of MOB_P_ type relaxases is indicated with a vertical arrow. (C) Samples of pGR16B with (RelLS20) or without (control) prior incubation with Rel_LS20_ were used as template DNA in sequencing reactions using forward primer 28 or reverse primer 29. The area of grey background, corresponding to the signal strength obtained with the control sample, is duplicated and overlaid to the same positions of the chromatogram of the Rel_LS20_-treated sample to highlight the drop in signal intensity. Note that the intensities of the signals are very similar for other regions. The residual signal observed after the drop in signal intensity is due to the presence of low amounts of non-nicked plasmid DNA in the sample.

Relaxases introduce a site- and strand-specific cut at the so-called *nic* site within the *oriT* region and remains covalently attached to the 5´ end via a tyrosine residue. This results in relaxation of the covalently closed circular form of the plasmid. To provide conclusive evidence that pLS20cat gene *58* encodes a relaxase we analyzed samples of the *oriT*_*LS20*_-containing plasmid pGR16B on an agarose gel after being incubated in a buffer with or without purified Rel_LS20_. As shown in Fig 4A, a fraction of the plasmid migrated to a higher position in the gel compared to the Rel_LS20_-untreated sample, showing that Rel_LS20_ had relaxed a portion of the plasmid molecules. Rel_LS20_ nicked pGR16B in a concentration dependent manner (S5A Fig), but it did not or hardly nicked the control plasmid pUCTA2501 lacking *oriT*_*LS20*_ (S5B Fig). Plasmids pGR16A and pGR16B gave similar nicking efficiencies, indicating that the nicking reaction was independent of the orientation of *oriT*_*LS0*_ (S5C Fig). Rel_LS20_ did not relax the plasmid, however, when the reaction mixture contained the chelating agent EDTA (S5D Fig), supporting the view that Rel_LS20_ requires divalent cation(s) like Mg for the nicking reaction, as has been observed for other relaxases containing a Histidine triad [19,24, for review see, 26].

Relaxases are often multidomain proteins. Without exception the relaxase domain is located in the N-terminal region of the protein. To test if this also applies to Rel_LS20_ we purified its N-terminal region (residues 1–232, which includes the predicted Histidine triad) fused to a His_(6)_ tag from *E*. *coli*, and used this protein, named N-Rel_LS20_, in nicking reactions. As shown in S5E Fig, N-Rel_LS20_ relaxed part of the *oriT*_*LS20*_ containing plasmid pGR16B, but not the parental vector pUCTA2501 lacking *oriT*_*LS20*_ indicating that the N-Rel_LS20_ domain contains nicking activity.

In all cases studied, the catalytic residue present in the relaxase domain that makes a transient 5´ phosphotyrosine bond with the nucleotide at the nick site corresponds to a tyrosine [for review see, 26]. Based on the following observation, residue Tyr26 might correspond to the catalytic tyrosine residue of Rel_LS20_. The catalytic Tyr residue of MOB_P_ family relaxases is present in MOB_P_ signature 5. MAST analyses showed that this MOB_P_ signature is conserved in 7% of the MOB_L_ relaxases (S3 Table). Rel_LS20_ is one of the MOB_L_ members containing the MOB_P_ signature 5. The position of the MOB_P_ signature 5 in Rel_LS20_ overlaps with that of MOB_L_ signature 5 (it is a coincidence that both signatures are named signature 5). An alignment of these two signatures suggests that the catalytic Tyr residue of MOB_P_ type relaxases corresponds to Rel_LS20_ residue Tyr26 and it shows that this tyrosine residue is almost fully conserved in the Rel_LS20_-related putative relaxases (see Fig 4B). To obtain evidence that Tyr26 is the catalytic tyrosine of Rel_LS20_ we engineered a mutant in which codon 26 encodes for a phenylalanine, and used the purified mutant, Rel_LS20_-Y26F, in nicking reactions. No substantial nicking activity was observed for the Y26F mutant (S5F Fig) supporting the view that Y26 is the active Tyrosine.

To locate the position of the nick site within *oriT*_*LS20*_, we prepared samples of pGR16B that were treated with or without Rel_LS20_ and subsequently used these in DNA sequencing reactions with convergently oriented primers 28 (forward) or 29 (reverse) that hybridize to regions flanking the *oriT*_*LS20*_ (Fig 4C). Very similar signal intensities were obtained in both reactions using the reverse primer 29. However, in the case of primer 28 the intensity of the signal dropped sharply in the Rel_LS20_-treated DNA sample after the sequence 5´-GAATCGGGGGCCGG-3´. Most likely this drop in signal intensity was due to a nick at this position in the template strand for primer 28.

The *nic* site is flanked by a 30 bp stretch of high GC content (73%) and coincides with the beginning of the inverted repeated sequence 5´-GGCTTTTTGCtgccGCAAAAAGCC-3´ (see Fig 2B). Thus, Rel_LS20_ introduces a nick at a position flanking an inverted repeat in the template strand of the conjugation genes. Implicitly, these results demonstrate that pLS20cat gene *58* encodes the relaxase Rel_LS20_. Fig 2A shows that the mobilization efficiency of the fragments endowing mobility to the pUCTA2501 derivative was 5 to 10 fold higher when cloned in orientation “B” compared to orientation “A”. The orientation of an *oriT* sequence determines the DNA strand that is transferred into the recipient cell, implying that one pUCTA2501 DNA strand is established better in the recipient than the other. As explained in the discussion, this observation suggests that Rel_LS20_ nicks the template strand of the conjugation genes, which is in line with the results presented in Fig 4C.

## Discussion

Here we have identified the *cis*-acting origin-of-transfer region *oriT*_LS20_ and the relaxase gene encoding the trans-acting Rel_LS20_ protein from pLS20. The relaxosome module is embedded within the large conjugation operon that encompasses genes *28* to *74* [17]. The results obtained have provided a better understanding of the relaxosome components present on Gram-positive mobile elements in general and particularly those belonging to the phylum Firmicutes.

### *oriT*_LS20_

We have delineated *oriT*_LS20_ to a region of 362 bp, which is intrinsically bent. Interestingly, about 10-fold higher mobilization efficiencies were obtained for pUCTA2501 derivatives in which *ori*T_LS20_ was cloned in orientation “B” compared to orientation “A” (see Table 2). Most probably this difference is due to the following. Relaxases cleave DNA at a site- and strand-specific position, and the nicked strand is transferred into the recipient cell. Consequently, the orientation of *oriT* dictates which DNA strand is transferred into the recipient cell. The replication protein of rolling-circle plasmids, which is related to relaxases, initiate a novel round of replication by introducing a site- and strand-specific nick in the plasmid at the so called double-strand origin (DSO). The 3´-end of the nicked site is used for DNA elongation, which is coupled to displacement of the replicated strand. To complete one round of replication the fully displaced circular ssDNA replication intermediate has to be converted into dsDNA. Efficient ss to dsDNA conversion starts at a specific region called single strand origin (SSO). Several families of SSOs are known, and they all have in common that they are only functional in only one orientation [15]. Since the orientation of *oriT* dictates the DNA strand that is transferred to the recipient cell, only in one orientation of *oriT* the transferred ssDNA strand will have the SSO in its functional orientation, resulting in optimal conversion of the ssDNA to double stranded plasmid DNA. The relation between functionality of an SSO and mobilization efficiency has been reported for other rolling-circle plasmids [34]. According to this reasoning, the *nic* site would be located in the template strand of the conjugation genes. This was indeed confirmed biochemically (Fig 4). *oriT´s* can be located at distinct positions with respect to the relaxase gene. In the case of pLS20cat the *oriT* and the relaxase are separated by a region of 865 bp containing two putative genes.

### The relaxase Rel_LS20_

The relaxase of pLS20cat was not identified by performing standard Blast searches. However, we did identify a stretch of 17 residues in the protein encoded by gene *58* that encompasses a putative HUH motif present in the superfamily of the so-called HUH endonucleases that includes relaxases [26]. We were able to show that gene *58* is required for conjugation, that Rel_LS20_ can act in *trans*, and we determined the Rel_LS20_-mediated strand and site-specific nick site within the *oriT*_LS20_ region. Together, these results show that gene *58* encodes the relaxase gene of pLS20cat, which we named *rel*_*LS20*_. We also provided evidence that the nicking activity resides in the N-terminal domain of Rel_LS20_ and that tyrosine 26 constitutes the catalytic residue responsible for making a transient 5´ phosphotyrosine bond with the nucleotide at the nick site.

### Rel_LS20_ is the prototype of a novel relaxase family, MOB_L_, that contains a large number of relaxases which are almost exclusively encoded in bacteria belonging to the phylum Firmicutes

By performing psiblast searches (7 iterations) we identified 817 sequences in public databases that may encode proteins showing significant homology with Rel_LS20_ (cutoff 1e-15), and we classified these relaxases into a new family named MOB_L_. Based on neighbor-joining analysis, the 817 (putative) relaxases can be subdivided into 2 clades (see S3 Fig) and a large fraction of the clade 1 *mob*_*L*_ relaxase genes are located on a plasmid.

Interestingly, four relaxases identified in our database screens as members of the MOB_L_ family, had been classified before in the MOB_P_ family. These four members concern NCBI reference accession numbers: WP_011264114.1 (encoded by plasmid pBCNF5603 of *Clostridium perfringens* F5603), WP_010968268.1 (encoded by plasmid pCP13 of *C*. *perfringens* strain 13), WP_011377372.1 (encoded by conjugative plasmid pHTβ of *E*. *faecium*) [30], and WP_012477521.1 (encoded by the 21 kb pEF1 plasmid of *E*. *faecium* 6T1a) [35]. When these relaxases were classified in 2009 it was mentioned that “*the phylogenetic position of these proteins is out of the clades of the MOB*_*P*_ *tree described in this work*” [24]. Therefore, we propose that these four relaxases belong to the MOB_L_ family defined here.

**Fig 5:**
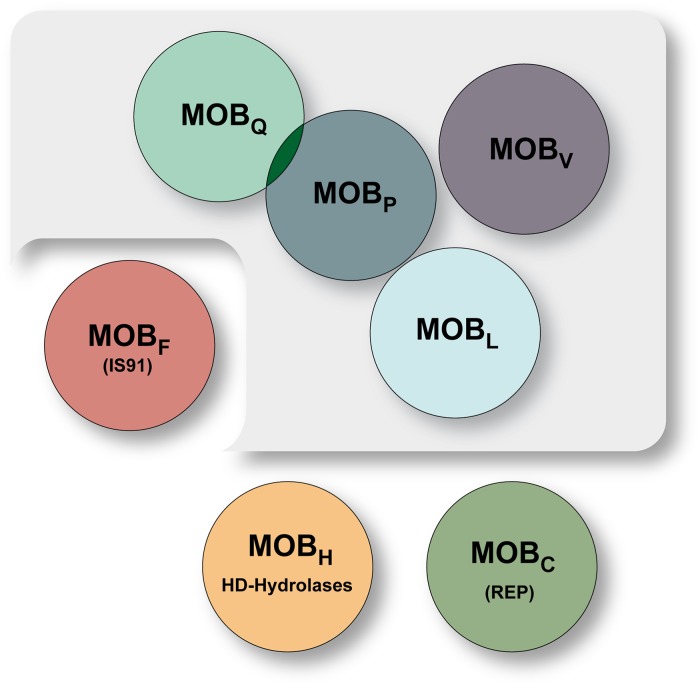
Schematic presentation of the relationships between the main relaxase protein families. The MOB_Q_, MOB_V_, MOB_P_, and MOB_L_ families belong to one large cluster (shown on a grey background). The other MOB families are divided into three independent groups. Note that these overlap with other protein families having DNA nicking-closing activities such as the replication proteins of rolling-circle plasmids (REP), IS91-like transposases (IS91) or HD hydrolases. The MOB_F_ family of relaxases contain a motif III that is also present in relaxases of the MOB_Q_, MOB_P_, MOB_V_ and MOB_L_ families. MOB_P_ includes relaxases that are also classified as MOB_HEN_.

Previously, a scheme depicting the relationship between the main relaxase families has been presented [24]. In this scheme the MOB_Q_, MOB_V_ and MOB_P_ families form a large cluster. The other MOB families, including protein families having nicking-closing activities that overlap with some of these MOB families, have been placed into three other groups. We identified Rel_LS20_ based on the presence of a putative 3-histidine motif, and sequences encompassing this motif (MOB_L_ signature 1) are highly conserved among the MOB_L_ relaxases. A 3-histidine motif is also present in relaxases belonging to the MOB_F_, MOB_Q_, MOB_V_ and MOB_P_ families. Using the Motif and Alignment tool MAST, sequences showing significant similarity to MOB_L_ signature 1 were identified in a number of relaxases of the MOB_Q_, MOB_V_ and MOB_P_ families where they overlap with the corresponding 3-histidine motif. However, MOB_L_ signature 1 does not show significant similarity with the 3-histidine motif of relaxases of the MOB_F_ family. This indicates that the MOB_L_ family is more closely related to the MOB_Q_, MOB_V_ and MOB_P_ families. Additional analyses revealed that the MOB_L_ family is most closely related to the MOB_P_ family. Based on this we propose the MOB_L_ family being a new member of the previously defined cluster including the MOB_Q_, MOB_V_ and MOB_P_ families. An updated scheme of the relationships between the main relaxase protein families is presented in Fig 5.

Relaxases belonging to the previously defined six MOB families are generally present in multiple bacterial phyla [24]. An important finding of this work is that the vast majority (98.2%) of the MOB_L_ relaxases are present in bacteria belonging to the phylum of Firmicutes. It is therefore likely that the MOB_L_ type relaxases play a prominent role in horizontal gene transfer in Firmicutes bacteria. The finding that many of the MOB_L_ relaxases identified here are located on plasmids supports this assumption.

Firmicutes bacteria can be found in a wide variety of habitats. One of the habitats in which Firmicutes are notably present is the gastrointestinal tract of mammals including humans, and evidence is accumulating that a balanced gut microbiome plays important roles in the health of these organisms [36]. Dysbiosis in the gut microbiome composition as a consequence of an oral antibiotic treatment can have substantial consequences [37]. The effects can be worsened when the gut microbiome contains bacteria that are resistant to the antibiotic treatment, especially when the resistant bacteria concern (opportunistic) pathogens. In the recent years evidence has been presented that the number of antibiotic resistance genes in the gut microbiome is increasing and that this pool of resistance genes may contribute to future emergence and spread of antibiotic resistance in (human) pathogens [5,6]. Conjugation is considered the major mechanism responsible for antibiotic resistance in general. Together, this warrants a better understanding of the conjugative elements present in the gut microbiome. Relaxases are essential for conjugation. Therefore, they form a target to interfere with conjugation mediated spread of antibiotic resistance [22,38]. Moreover, once the essential relaxase gene has been identified the related conjugative element can be relatively easily recognized and studied. Therefore, the identification in this work of more than 800 (putative) relaxase genes present almost exclusively in bacteria of the phylum Firmicutes is likely to have significant impacts on the identification and analysis of mobile elements in this phylum of bacteria that is largely represented in mammalian gut microbiota.

## Materials and methods

### Bacterial strains, plasmids, media and oligonucleotides

*Escherichia coli* and *B*. *subtilis* strains were grown in Luria-Bertani (LB) liquid medium or on 1.5% LB agar plates. When appropriate, media were supplemented with the following antibiotics: ampicillin (100 μg/ml), erythromycin (1 and 150 μg/ml in *B*. *subtilis* and *E*. *coli*, respectively), chloramphenicol (5 μg/ml), spectinomycin (100 μg/ml), and kanamycin (10 and 30 μg/ml in *B*. *subtilis* and *E*. *coli*, respectively). *B*. *subtilis* strains used were isogenic with *B*. *subtilis* strain 168 and are listed in S4 Table. Plasmids and oligonucleotides used are listed in S5 and S6 Tables, respectively. All oligos were purchased from Isogen Life Science, The Netherlands.

### Transformation

*E*. *coli* cells were transformed using standard methods [39]. Preparation of competent *B*. *subtilis* cells and transformation were carried out as described before [40]. Transformants were selected on LB agar plates with appropriate antibiotics. pLS20cat encodes a protein, Rok_LS20_, that inhibits the development of competence by repressing *comK*, the key transcriptional activator of competence genes [41]. Therefore, to manipulate genes on pLS20cat we prepared competent cells of a pLS20cat-harboring strain that contains a chromosomal P_xyl_-*comK* fusion (PKS56) using a standard protocol [41].

### Construction of plasmids and strains

The correctness of sequences of all cloned PCR fragments was confirmed by sequence analysis. Amplification by PCR of pLS20cat regions was performed using total DNA isolated from pLS20cat harboring strain PKS11 as template. Plasmid pLS20Δ56–58 was constructed by replacing genes *56*, *57* and *58* with the kanamycin gene of plasmid pBEST501 as follows. The upstream region of gene *56* (PCR-UP-56) and the downstream region of gene *58* (PCR-DN-58) were amplified by PCR using pLS20cat as template DNA in combination with primer sets oGR56/oGR57 and oGR58/oGR59, respectively. The resulting PCR fragments were digested with *Bam*HI and *Sal*I. Plasmid pBEST501 was digested with *Bam*HI and *Sal*I and the resulting 800 bp fragment containing the kanamycin gene was purified. Next, the DNA fragments corresponding to PCR-UP-56, PCR-DN-58, and the kanamycin gene were used to prepare a ligation mixture. After ligation, this mixture was used to transform competent cells of pLS20cat-containing *B*. *subtilis* cells of strain PKS56. Deletion of genes *56–58* of kanamycin-resistant transformants was first confirmed by PCR using primers Ori-UP and Ori-Dn. Next, the absence of mutations in the PCR amplified regions was confirmed by sequence analysis, which in addition affirmed replacement of genes *56–58* by the kanamycin resistance gene. The resulting strain containing plasmid pLS20Δ56–58 was named GR148. Finally, pLS20Δ56–58 was isolated from strain GR148 and used to transform competent cells of the appropriate strain.

The following strategy was used to clone the pLS20cat genes *56*, *57* and *58* behind the IPTG-inducible P_*spank*_ promoter in the *B*. *subtilis amyE* integration vector pDR110. The genes were amplified by PCR using primers oGR43 and oGR60. After purification, the PCR fragment was digested with *Nhe*I and *Sph*I and then ligated with the vector pDR110 cut with the same enzymes. Plasmid DNA of the constructed vector pGR27 was isolated from *E*. *coli* cells and then used to transform competent *B*. *subtilis* cells. Double-crossover integration into the chromosome was checked by the loss of amylase activity. A similar strategy was used to construct a *B*. *subtilis* strain GR206 in which pLS20cat genes *56* and *57* were placed under the control of the P_*spank*_ promoter at the chromosomal *amyE* locus. In this case, the genes were amplified by PCR using primers oGR27 and oGR133. The purified PCR fragment was digested with *SpeI* and *Sph*I and then ligated with the vector pDR110 cut with *Nhe*I and *Sph*I. Plasmid DNA of the constructed vector pGR52 was isolated from *E*. *coli* cells and then used to transform competent *B*. *subtilis* cells. Double-crossover integration into the chromosome was checked by the loss of amylase activity.

The *E*. *coli*/*B*. *subtilis* shuttle vector pUCTA2501 contains the replication functions of the cryptic *B*. *subtilis* rolling-circle plasmid pTA1015 [15] as well as the erythromycin resistance gene of pE194. The following strategy was used to create pUCTA2501 derivatives containing a fragment of plasmid pLS20cat. First, the desired pLS20cat region was amplified by PCR using appropriate primers. Next, after the purified PCR fragments had been digested with *XbaI*, the fragments were ligated to the vector pUCTA2501 linearized with *Xba*I, and the resulting ligation mixture was used to transform competent *E*. *coli* XL1-blue cells. The correctness and the orientation of the inserts were checked by sequencing. The names of the pUCTA2501 derivatives and their characteristics are listed in S5 Table. Each pUCTA2501 derivative plasmid was then used to transform competent *B*. *subtilis* 168 cells. The presence of the plasmid in erythromycin-resistant transformants was confirmed by PCR and pLS20cat was introduced into the strain by conjugation using strain PKS11 as donor. The resulting strains are listed in S4 Table.

The pLS20cat gene 58 (*rel*_*LS20*_) was cloned as follows in the *E*. *coli* expression vector pET28b+ to generate a fusion gene containing a C-terminal *his*_*(6)*_ extension. *Rel*_*LS20*_ was amplified with primerset oWM001-oWM002. The resulting PCR fragment was digested with *Nco*I and *Sal*I and cloned in pET28b+ digested with the same restriction enzymes. The resulting pET28b+ derivative named pAND83, was constructed using *E*. *coli* strain XL-blue1. Once verified its correctness, the plasmid was transformed into *E*. *coli* strain BL21(DE3). Using the same strategy, the N-terminal region of pLS20cat gene 58 (*N-rel*_*LS20*_) was cloned in the pET28b+ vector. In this case, *N-rel*_*LS20*_ was amplified with primerset 0WM001-oWM003. The derivative of *rel*_*LS20*_ in which Tyrosine codon 26 was mutated to encode a Phenylalaline (*rel*_*LS20*_-*Y26F*) was constructed as follows. A DNA fragment containing the desired mutation was generated by PCR using pAND83 as template and primerset oWM001A-oWM002. The resulting DNA fragment was then extended at its 5´ end by two PCR reactions. First, the PCR product was used as template in a PCR reaction with primerset oWM001B-oWM002; and second, this PCR product served as template for the final PCR reaction using primerset oWM001-oWM002. Next, the PCR fragment was cloned into pET28b+ using the same strategy as that used for cloning the wild type *rel*_*LS20*_.

### Conjugation/mobilization assays

Conjugation was carried out in liquid medium as described previously [17]. The effect of ectopic expression of a given gene placed under the control of the inducible P_*spank*_ promoter on conjugation was studied by adding the inducer (1 mM IPTG) to prewarmed LB medium used to dilute overnight cultures of the donor cells.

### Analytical ultracentrifugation experiments

#### Sedimentation velocity assays (SV)

Samples at concentrations ranging from 12 to 48 μM, in 20 mM Tris, 500 mM NaCl, 10 mM MgCl_2_, 1 mM EDTA, 0.1 mM β-Mercaptoethanol and 1% glycerol, pH 7.4, were loaded (320 μL) into analytical ultracentrifugation cells. The experiments were carried out at 48,000 rpm in a XL-I analytical ultracentrifuge (Beckman-Coulter Inc.) equipped with both UV-VIS absorbance and Raleigh interference detection systems, using an An-50Ti rotor, and 12 mm Epon-charcoal standard double-sector centerpieces. Sedimentation profiles were recorded at 230 nm. Differential sedimentation coefficient distributions were calculated by least-squares boundary modelling of sedimentation velocity data using the continuous distribution *c*(*s*) Lamm equation model as implemented by SEDFIT [42]. These *s* values were corrected to standard conditions (water, 20°C, and infinite dilution) using the program SEDNTERP [43] to obtain the corresponding standard *s* values (*s*_20,*w*_).

#### Sedimentation equilibrium assays (SE)

SE experiments of Rel_LS20_ samples were carried out using short columns (100 μL) at speeds ranging from 12,000 to 15,000 rpm and at two wavelengths (230 and 280 nm), using the same experimental conditions and instrument as in the SV experiments. After the last equilibrium scan, a high-speed centrifugation run (48,000 rpm) was done to obtain the corresponding baseline offsets. Weight-average buoyant molecular weights of protein were determined by fitting a single species model to the experimental data using the HeteroAnalysis program [44] and corrected for solvent composition and temperature with the program SEDNTERP [43].

#### Dynamic light scattering assays (DLS)

DLS experiments were carried out in a Protein Solutions DynaPro MS/X instrument (Protein Solutions, Piscataway, NJ) at 20°C using a 90° light scattering cuvette. Prior to measurements, samples at the same experimental conditions used in SV and SE, were centrifuged for 10 min at 12,000g and 4°C. Data were collected and analyzed with Dynamics V6 Software.

#### Estimate of molar mass of Rel_LS20_ from hydrodynamic measurements

The apparent molar mass of a single sedimenting solute species (*M*) may be calculated using measured values of the sedimentation coefficient *s* and the diffusion coefficient *D* according to the Svedberg equation [45],

$$M=\frac{RTs}{(1-\bar{\nu }\rho )D}$$

where *T*, *R*, and *ρ* stand for the absolute temperature, the universal gas constant and the density of the solution, respectively. The estimate of molar mass obtained via this relation is independent of the shape of the sedimenting/diffusing species, as frictional coefficients for sedimentation and diffusion cancel in the derivation of the equation.

### Overexpression and purification of recombinant Rel_LS20_ and derivatives containing a C-terminal His_(6)_ tag

A recombinant version of Rel_LS20_ was expressed and purified as follows. *E*. *coli* BL21(DE3) cells containing plasmid pAND83 (*rel*_*LS20*_*His*_*(6)*_) were inoculated in fresh LB media complemented with 30 μg/ml kanamycin and grown at 37°C with shaking (200 rpm). At an OD_600_ of about 0.6, IPTG was added to a final concentration of 1 mM to induce the recombinant protein and growth was continued for 2 h. Next, cells were collected by centrifugation and processed as described previously [41]. The nickel-column purified proteins (>95% pure) were finally dialysed against buffer B (20 mM Tris-HCl pH 8.0, 1 mM EDTA, 500 mM NaCl, 10 mM MgCl_2_, 7mM β-mercaptoethanol, 50% v/v glycerol) and stored in aliquots at -80°C. Bradford assay and OD_280_ determination were used to determine the protein concentrations. The SDS PAGE presented in S6 Fig visualizes the purification step of Rel_LS20_. The same methodology was used to purify the N-terminal domain of Rel_LS20_ and the Y26F mutant protein. Apparently, the Tyr26F mutation affects the stability of the protein. This conclusion is based on our findings that the yield of purified mutant protein was much lower than that obtained for full-length Rel_LS20_ or its N-terminal domain. Consequently, the level of purity obtained for the mutant protein was lower than that for the full-length Rel_LS20_ or N-Rel_LS20_.

### Determination of the Rel_LS20_-mediated nick site in *oriT*_*LS20*_

Rel_LS20_ nicking activity was assayed in 10 μL reaction volumes containing 2 nM supercoiled plasmid pGR16B and the indicated protein concentration in 20 mM Tris/HCl pH 7.5, 5 mM MgCl_2_, 5 mM NaCl, 0.1 mM EDTA and 50 μg/mL BSA. The reaction mixture was incubated for 15 minutes at 37°C in a water bath. To stop the reaction, 25 μg/mL Proteinase K, 0.5% SDS and 25mM EDTA were added followed by incubation at 37°C for 20 minutes. Then, 6X DNA loading buffer was added to a final concentration of 1X, and all the volume was loaded in a 0.8% agarose gel, prestained with Sybr-safe. Gels were run at 20V overnight at 4°C and then photographed. In addition, nicking reactions were carried out in duplicates, and after the reactions were stopped the DNA was purified using a commercial kit (Wizard, Promega) after which the purified plasmid DNAs were employed in Sanger sequencing reactions using primer oGR28 or oGR29.

### *In silico* analyses

#### Prediction of static bents in pLS20cat DNA

The entire pLS20cat sequence (accession number NC_015148.1) was analyzed for the presence of intrinsic bents according to the dinucleotide wedge model using parameters “AAWedge” and “Cacchione & De Santis” [46] implemented in the program *dnacurve*.*py (*http://www.lfd.uci.edu/~gohlke/code/dnacurve.py.html*)*. To allow analysis of the entire plasmid the default sequence length limit of the program was raised. Two set of parameters were tested (“AAWedge” and “Cacchione & De Santis”). For both, the curvature, and the curvature-angle were calculated for each position using window sizes of 10 and 15 bp, respectively. These values were smoothed using a sliding window of 600 bp and normalized to mean 0 and standard deviation 1.

#### Identification of Mob_L_ members

Rel_LS20_ was used as a query sequence to execute a psi-blast search against the NCBI nr protein database (version 2.2.30+, January 17, 2015), allowing up to 10 rounds of reiteration with and E-value threshold of 1e-15 [47–49]. This search resulted in the detection of 962 sequences. The program “USEARCH” (version 8.0.1517_i86linux32) was then used to identify and remove redundant sequences showing 100% identity [50], resulting in 817 unique hits showing high similarity to Rel_LS20_.

#### MEME and MAST

The motif-identification program MEME (Bailey and Elkan, 1994) (http://meme-suite.org/tools/meme, version 4.10.0) was used to identify conserved motifs, which we named “signatures” to distinguish them from “motifs” published before, in members of the MOB_L_ and previously described MOB families [24]. We noted that more specific motifs were obtained when reducing the redundancy between the input sequences. The function “cluster_fast” of the program USEARCH [50] was used to remove similar sequences, keeping only the centroids of clusters of sequences that shared more than 50% of identity. MEME was run against the remaining proteins, setting the search to retrieve up to a maximum of ten motifs for each family with an E-value below 1e-15. The program MAST [51] of the MEME suite was used to study if an identified motif of a given MOB family was present in member(s) of any of the other MOB families using an E-value threshold of 1e-6.

#### Neighbor-joining analysis

The program “MEGA6” [52] was employed for constructing a condensed phylogenetic tree by reducing the length of interior branches with low statistical support to 0. Protein sequences longer than 300 residues were included in the analysis. The phylogeny was built by neighbor-joining and tested by 1000 bootstraps.

#### Crossing MOB_L_ members against constructed plasmid database

Plasmids deposited in the NCBI nr database were retrieved by screening the annotations for the keywords “plasmid” and “circular DNA”. The 10,904 plasmids retrieved at 27 May 2015 were used to build a blast database. Next, each MOB_L_ member was run against the constructed plasmid database using tblastn. A MOB_L_ member was considered to be located on a plasmid if an identity of more than 95% was identified over more than 80% of the entire protein sequence.

## Supporting information

S1 Fig Identification of ten signatures in MOB_L_ type relaxases and distribution of the identified signatures in the primary sequence of Rel_LS20_. (A) Weblogo representation of the ten signatures identified in the MOB_L_ type relaxases using the motif-identification program MEME. Name of the signatures (sig) is given on the left. The position of the signature in the primary Rel_LS20_ sequence is presented at the right. (B) Position of ten signatures identified by MEME for the MOB_L_ type relaxases in the Rel_LS20_ primary sequence. The primary sequence of the Rel_LS20_ protein is presented along with the position of eight MOB_L_ signatures identified by MEME. The colour code used for the signatures is consistent with that used for the Weblogo presentations given in (A). The Rel_LS20_ region showing similarity with part of the motifs III of the MOB_P_ MOB_Q_ and MOB_V_ type relaxases (see Fig 1) is highlighted in red. Note that MOB_L_ signatures III and IX are not present in Rel_LS20_.
(TIF)

S2 Fig Weblogo presentation of up to ten signatures identified for relaxases belonging to the MOB_P_ (A), MOB_Q_ (B), MOB_V_ (C), MOB_F_ (D), MOB_H_ (E), and MOB_C_ (F) family using the motif-identification program MEME. Names of the signatures (sig) is given on the left.
(PDF)

S3 Fig Phylogenetic tree of MOB_L_ family of relaxases. The phylogenetic tree was constructed using the program “MEGA6” (see Materials and methods) and phylogeny was built by neighbor-joining and tested by 1000 bootstraps. Members of the resulting two clades are given in red (clade 1) and blue (clade 2). Rel_LS20_, belonging to clade 1, is highlighted in green.
(PDF)

S4 Fig *In silico* analyses predicts a static bent in the pLS20cat region near *oriT*_*LS20*_. The entire pLS20cat sequence (accession number NC_015148.1) was analyzed for the presence of intrinsic bents according to the dinucleotide wedge model [46, see Materials and methods]. The predicted probability of sequences to form a static bent is presented as a function of the pLS20cat sequence. Maximum values peak around pLS20cat positions 2,200 and 27,500. The latter position, -27,500-, coincides with *oriT*_*LS20*_. Positions around 2,200 correspond to the region containing the divergently oriented promoters P_rco_ and P_c_ driving expression of regulatory gene *rco*_*LS20*_ and the conjugation operon, respectively, which has been demonstrated to contain a static bent [18].
(TIF)

S5 Fig Biochemical characterization of Rel_LS20_. (A) The efficiency of the Rel_LS20_ nicking reaction is concentration dependent. Plasmid pGR16B (2 nM) was incubated without (-) or with increasing amounts of Rel_LS20_ (4.25, 12.5, 25 and 50 nM, respectively). (B) A supercoiled plasmid lacking *oriT*_*LS20*_ is not relaxed by Rel_LS20_. The empty pUCTA2501 *oriT*-screening vector (2 nM), was incubated without (-) or with (+, 25 nM). (C) Rel_LS20_-mediated plasmid relaxation is independent of *oriT*_*LS20*_ orientation. Plasmids pGRA16A and pGRA16B (2 nM), which differ only in the orientation of *oriT*_*LS20*_, were treated without (-) or with (+, 25 nM) Rel_LS20_. (D) Rel_LS20_-mediated nicking is inhibited by the chelating agent EDTA. Plasmid pGR16B (2 nM) was treated with Rel_LS20_ (25 nM) in the absence (-) or presence (+) of 10 mM EDTA. (E) The nicking activity of Rel_LS20_ resides in its N-terminal domain. Plasmid pGR16B (2 nM) was incubated without (-) or with increasing concentrations of N-Rel_LS20_ (12.5, 25, 50, 100, 200 and 400 nM, respectively). In the last lane, labelled “C”, the empty vector pUCTA2501 was incubated with 200 nM N-Rel_LS20_. (F) Rel_LS20_ residue Tyr26 is important for nicking activity. Plasmid pGR16B (2 nM) was incubated without (-) or with Rel_LS20_Y26F (50 nM). After incubation, the samples were treated with proteinase K and the DNAs were separated on 0.8% agarose gels. The positions of supercoiled (sc) and nicked open circular DNA (oc) are indicated.
(TIF)

S6 Fig Purification of Rel_LS20_. A Coomassie Brilliant Blue stained 12% SDS PAA gel reflecting Rel_LS20_ nickel column purification steps. Rel_LS20_ was overexpressed in the *E*. *coli* strain AZ43 which corresponds to strain BL21(DE3) harboring pET28b+ derivative pAND84 (see S4 Table). Aliquots of different steps were loaded. Control lane “C”, 4 μl prestained marker proteins (ThermoFisher PageRuler Plus (10–250 kDa); increasing molecular weights of approximately 10, 15, 25, 35, 55, 70, 100, 130 and 250 kDa, respectively). Lane 1, supernatant fraction of induced AZ43 cells (5 μg total protein loaded). Lane 2, flow-through of the centrifuged total lysate adjusted to 20 mM imidazole after passing the Nickel column (5 μg total protein loaded). Lane 3, washing step (fraction 7, corresponding to ~40 mM imidazole (6 μl)). Lane 4, fraction 40, corresponding to ~100 mM imidazole (16 μl). Lane 5, pool of eluted fractions (lateral 200 mM imidazole elution peaks corresponding to fractions 12–15 and 19–40, 5 μg loaded). Lane 6, pool of eluted fractions (central 200 mM imidazole elution peak with highest concentrations of Rel_LS20_ corresponding to fractions 16–18, 5 μg loaded). Each fraction corresponded to 1 ml. The N-terminal domain of Rel_LS20_ and the Y26F mutant were purified using the same methodology.
(TIF)

S1 Table Proteins in the NCBI database showing significant similarity with Rel_LS20_. (PDF)

S2 Table Occurrence (%) of MOB_L_ signatures in members of other MOB families (%). (DOCX)

S3 Table Occurrence (%) of the signatures of the MOB_Q_, MOB_V_, MOB_P_, MOB_F_, MOB_C_ and MOB_H_ in members of the MOB_L_ family. (DOCX)

S4 Table Strains used. (DOCX)

S5 Table Plasmids used. (DOCX)

S6 Table Oligonucleotides used. (DOCX)
