# Supplementary figures and images for: Discovery of a new family of relaxases in Firmicutes bacteria
Source: PLoS Genet. 2017 Feb 16;13(2):e1006586. doi: 10.1371/journal.pgen.1006586 (PMC5313138; doi:10.1371/journal.pgen.1006586)

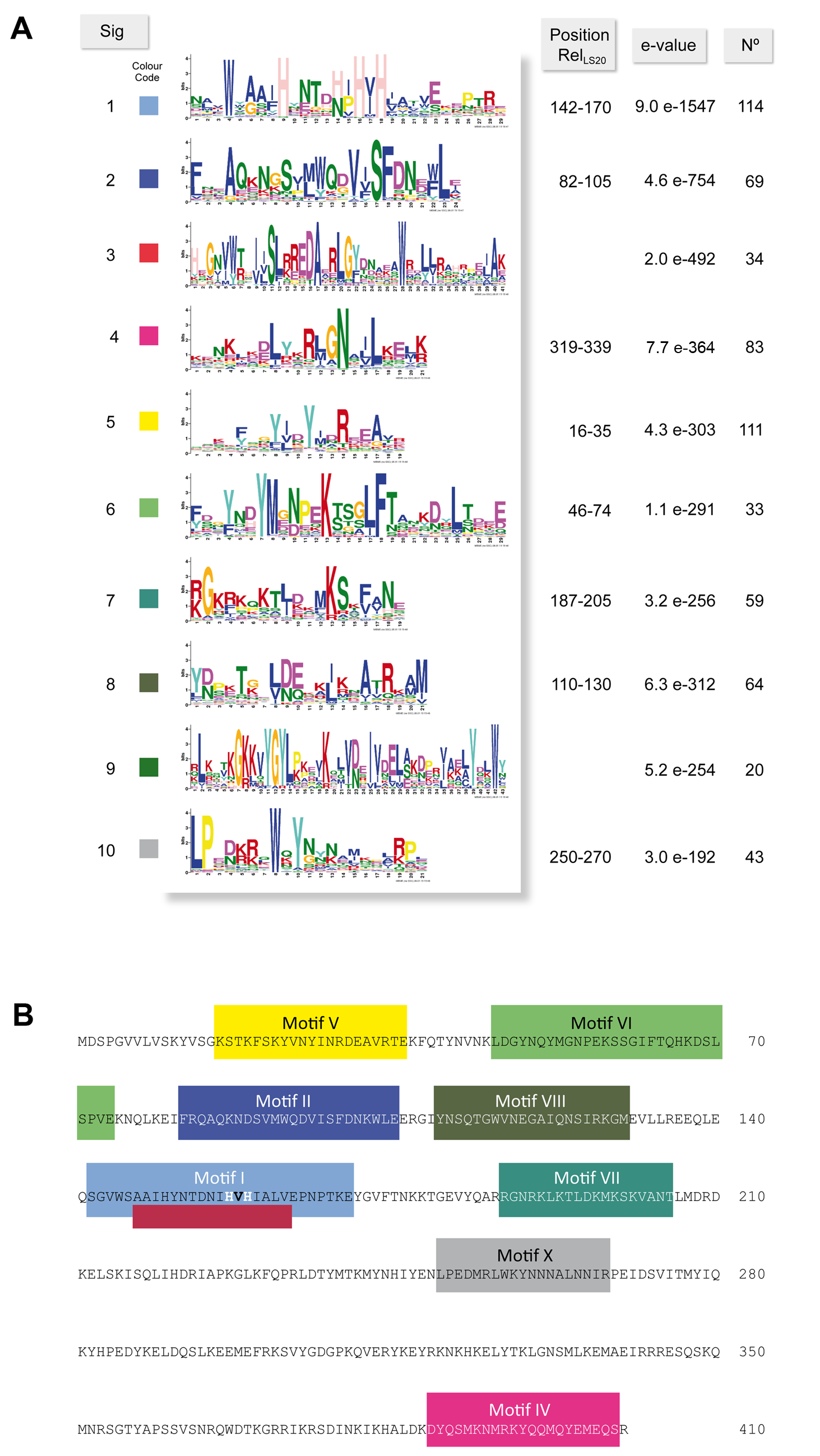

Supplement: S1 Fig — (A) Weblogo representation of the ten signatures identified in the MOBL type relaxases using the motif-identification program MEME. Name of the signatures (sig) is given on the left. The position of the signature in the primary RelLS20 sequence is presented at the right. (B) Position of ten signatures identified by MEME for the MOBL type relaxases in the RelLS20 primary sequence. The primary sequence of the RelLS20 protein is presented along with the position of eight MOBL signatures identified by MEME. The colour code used for the signatures is consistent with that used for the Weblogo presentations given in (A). The RelLS20 region showing similarity with part of the motifs III of the MOBP MOBQ and MOBV type relaxases (see Fig 1) is highlighted in red. Note that MOBL signatures III and IX are not present in RelLS20. (TIF) [file pgen.1006586.s001.tif]

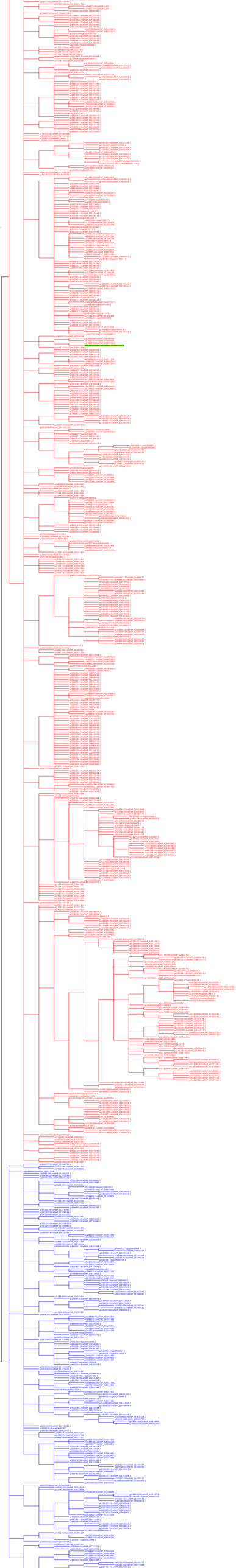

Supplement: S3 Fig — The phylogenetic tree was constructed using the program “MEGA6” (see Materials and methods) and phylogeny was built by neighbor-joining and tested by 1000 bootstraps. Members of the resulting two clades are given in red (clade 1) and blue (clade 2). RelLS20, belonging to clade 1, is highlighted in green. (PDF) [file pgen.1006586.s003.pdf]

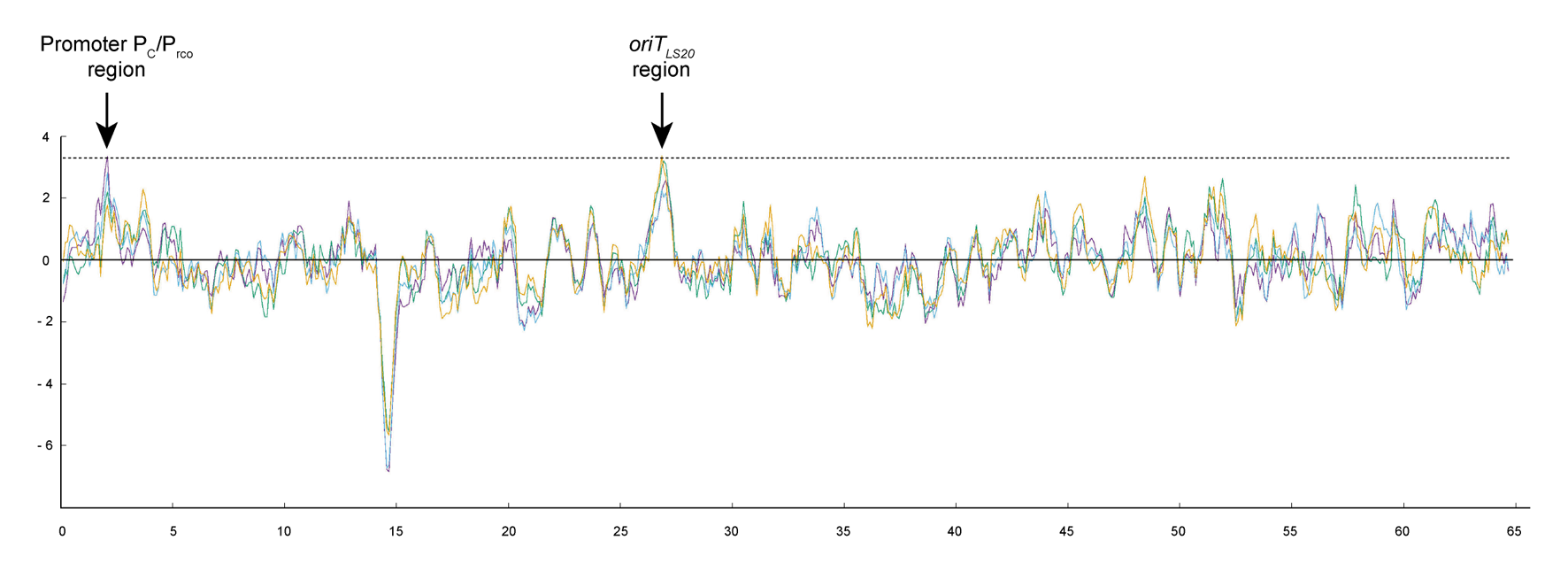

Supplement: S4 Fig — The entire pLS20cat sequence (accession number NC_015148.1) was analyzed for the presence of intrinsic bents according to the dinucleotide wedge model [46, see Materials and methods]. The predicted probability of sequences to form a static bent is presented as a function of the pLS20cat sequence. Maximum values peak around pLS20cat positions 2,200 and 27,500. The latter position, -27,500-, coincides with oriTLS20. Positions around 2,200 correspond to the region containing the divergently oriented promoters Prco and Pc driving expression of regulatory gene rcoLS20 and the conjugation operon, respectively, which has been demonstrated to contain a static bent [18]. (TIF) [file pgen.1006586.s004.tif]

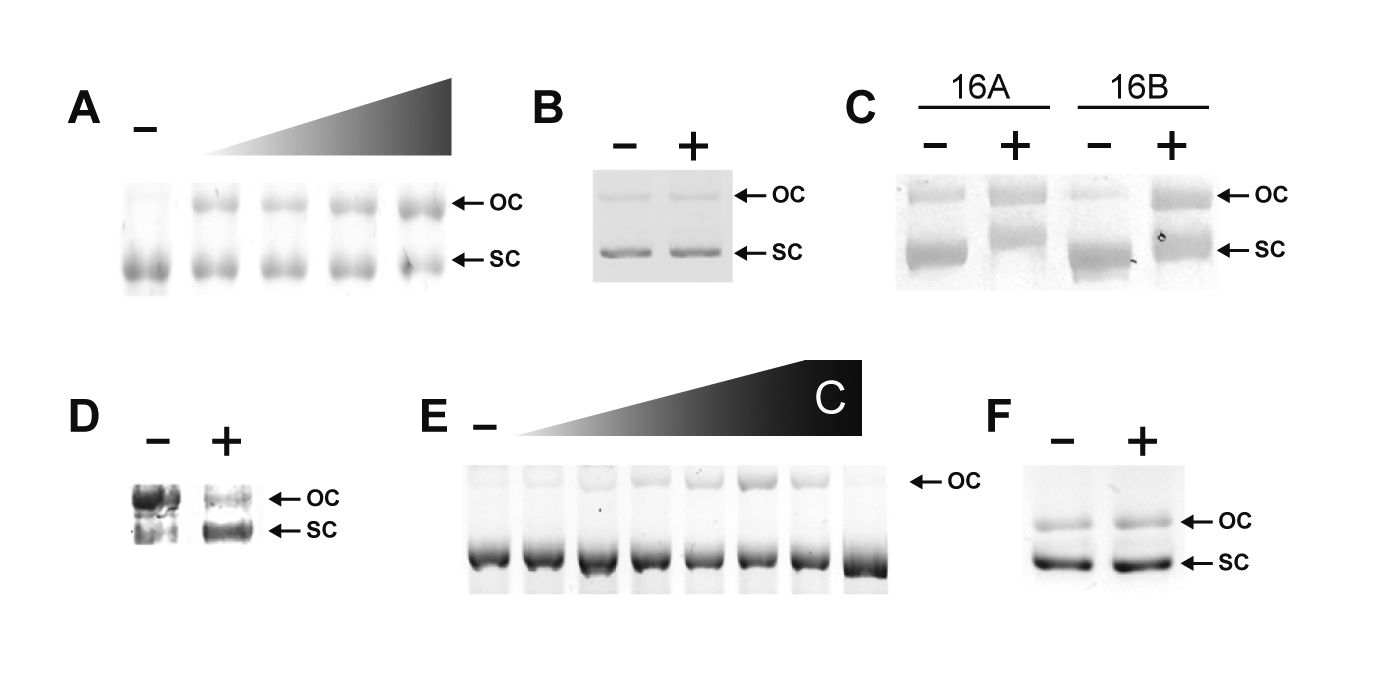

Supplement: S5 Fig — (A) The efficiency of the RelLS20 nicking reaction is concentration dependent. Plasmid pGR16B (2 nM) was incubated without (-) or with increasing amounts of RelLS20 (4.25, 12.5, 25 and 50 nM, respectively). (B) A supercoiled plasmid lacking oriTLS20 is not relaxed by RelLS20. The empty pUCTA2501 oriT-screening vector (2 nM), was incubated without (-) or with (+, 25 nM). (C) RelLS20-mediated plasmid relaxation is independent of oriTLS20 orientation. Plasmids pGRA16A and pGRA16B (2 nM), which differ only in the orientation of oriTLS20, were treated without (-) or with (+, 25 nM) RelLS20. (D) RelLS20-mediated nicking is inhibited by the chelating agent EDTA. Plasmid pGR16B (2 nM) was treated with RelLS20 (25 nM) in the absence (-) or presence (+) of 10 mM EDTA. (E) The nicking activity of RelLS20 resides in its N-terminal domain. Plasmid pGR16B (2 nM) was incubated without (-) or with increasing concentrations of N-RelLS20 (12.5, 25, 50, 100, 200 and 400 nM, respectively). In the last lane, labelled “C”, the empty vector pUCTA2501 was incubated with 200 nM N-RelLS20. (F) RelLS20 residue Tyr26 is important for nicking activity. Plasmid pGR16B (2 nM) was incubated without (-) or with RelLS20Y26F (50 nM). After incubation, the samples were treated with proteinase K and the DNAs were separated on 0.8% agarose gels. The positions of supercoiled (sc) and nicked open circular DNA (oc) are indicated. (TIF) [file pgen.1006586.s005.tif]

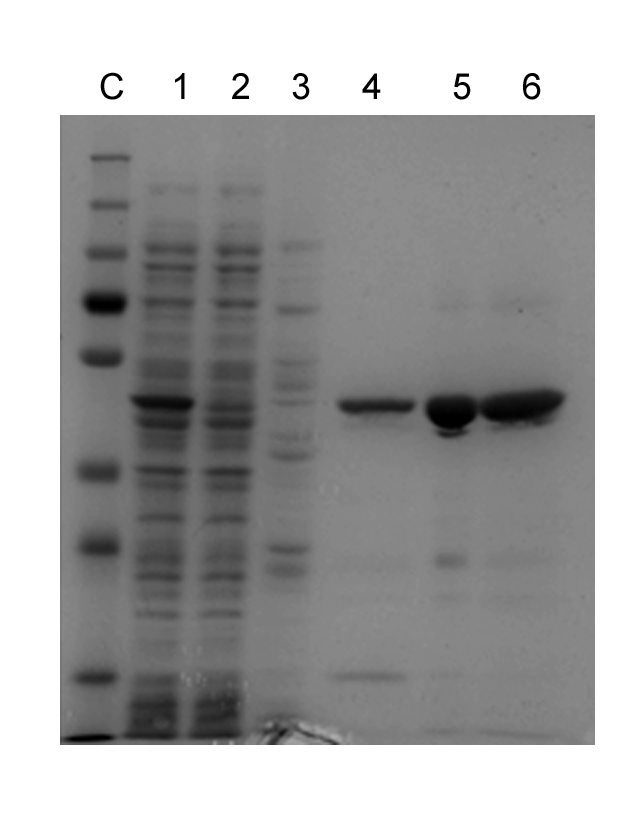

Supplement: S6 Fig — A Coomassie Brilliant Blue stained 12% SDS PAA gel reflecting RelLS20 nickel column purification steps. RelLS20 was overexpressed in the E. coli strain AZ43 which corresponds to strain BL21(DE3) harboring pET28b+ derivative pAND84 (see S4 Table). Aliquots of different steps were loaded. Control lane “C”, 4 μl prestained marker proteins (ThermoFisher PageRuler Plus (10–250 kDa); increasing molecular weights of approximately 10, 15, 25, 35, 55, 70, 100, 130 and 250 kDa, respectively). Lane 1, supernatant fraction of induced AZ43 cells (5 μg total protein loaded). Lane 2, flow-through of the centrifuged total lysate adjusted to 20 mM imidazole after passing the Nickel column (5 μg total protein loaded). Lane 3, washing step (fraction 7, corresponding to ~40 mM imidazole (6 μl)). Lane 4, fraction 40, corresponding to ~100 mM imidazole (16 μl). Lane 5, pool of eluted fractions (lateral 200 mM imidazole elution peaks corresponding to fractions 12–15 and 19–40, 5 μg loaded). Lane 6, pool of eluted fractions (central 200 mM imidazole elution peak with highest concentrations of RelLS20 corresponding to fractions 16–18, 5 μg loaded). Each fraction corresponded to 1 ml. The N-terminal domain of RelLS20 and the Y26F mutant were purified using the same methodology. (TIF) [file pgen.1006586.s006.tif]
