# Supplementary material for: Discovery of a new family of relaxases in Firmicutes bacteria
Source: PLoS Genet. 2017 Feb 16;13(2):e1006586. doi: 10.1371/journal.pgen.1006586 (PMC5313138; doi:10.1371/journal.pgen.1006586)
Supplement: S2 Fig — Names of the signatures (sig) is given on the left. (PDF) [file pgen.1006586.s002.pdf]

A

Signatures MOB_p family

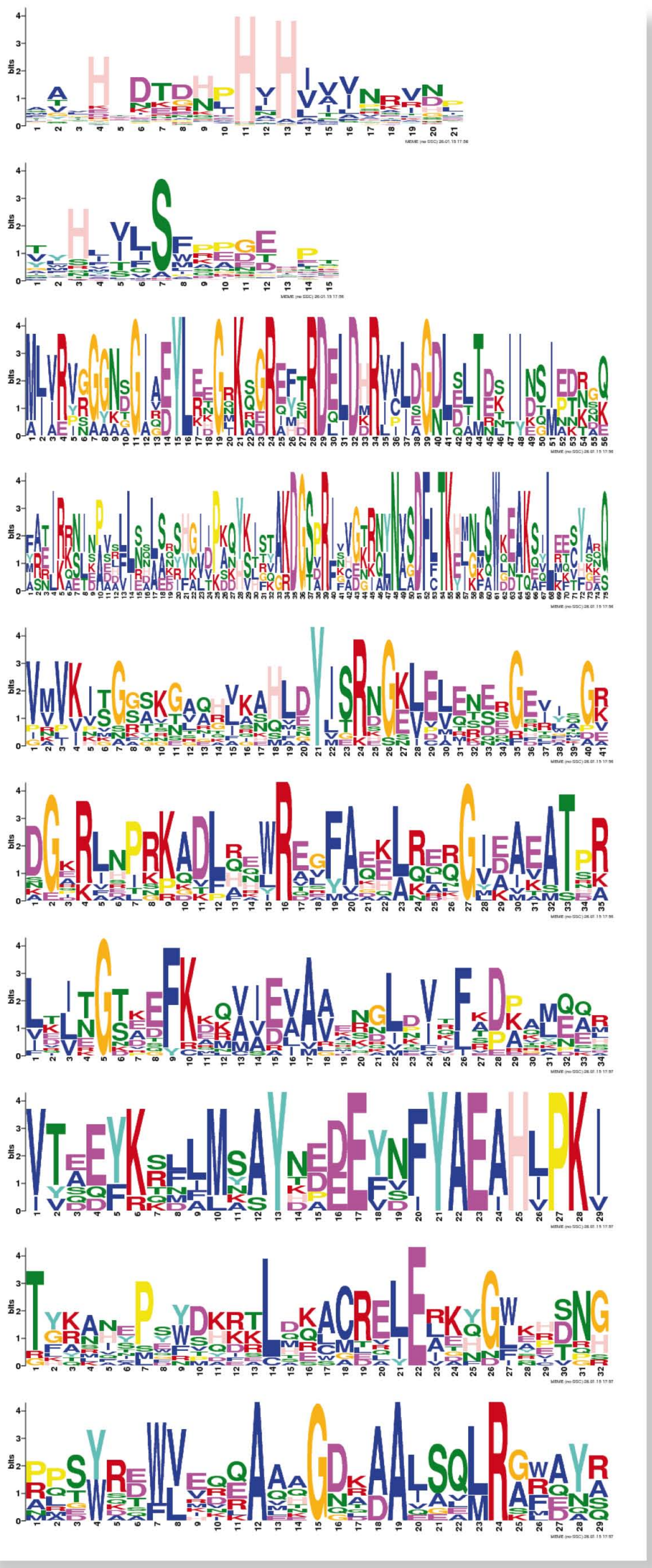

Sig	e-value	Nº
1	8.0 e-323	70
2	2.3 e-129	62
3	5.4 e-81	7
4	1.1 e-66	6
5	2.1 e-57	12
6	2.3 e-70	12
7	6.9 e-56	14
8	3.5 e-55	7
9	1.0 e-52	12
10	1.2 e-48	11

B Signatures MOB_Q family

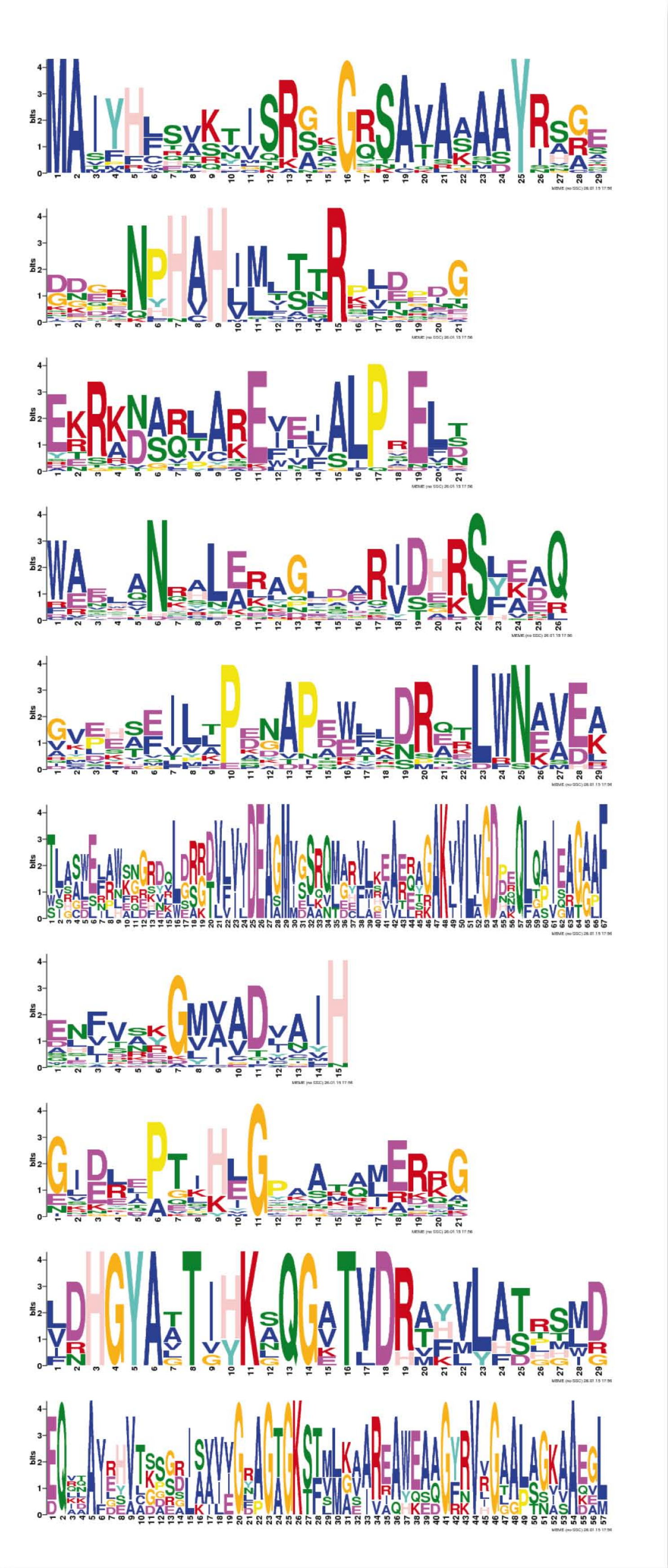

Sig	e-value	Nº
1	1.1 e-194	19
2	5.2 e-141	24
3	3.9 e-134	24
4	3.8 e-137	24
5	3.7 e-98	16
6	5.6 e-82	7
7	2.6 e-68	23
8	3.4 e-56	21
9	5.6 e-56	7
10	9.5 e-69	7

C

Signatures MOB_V family

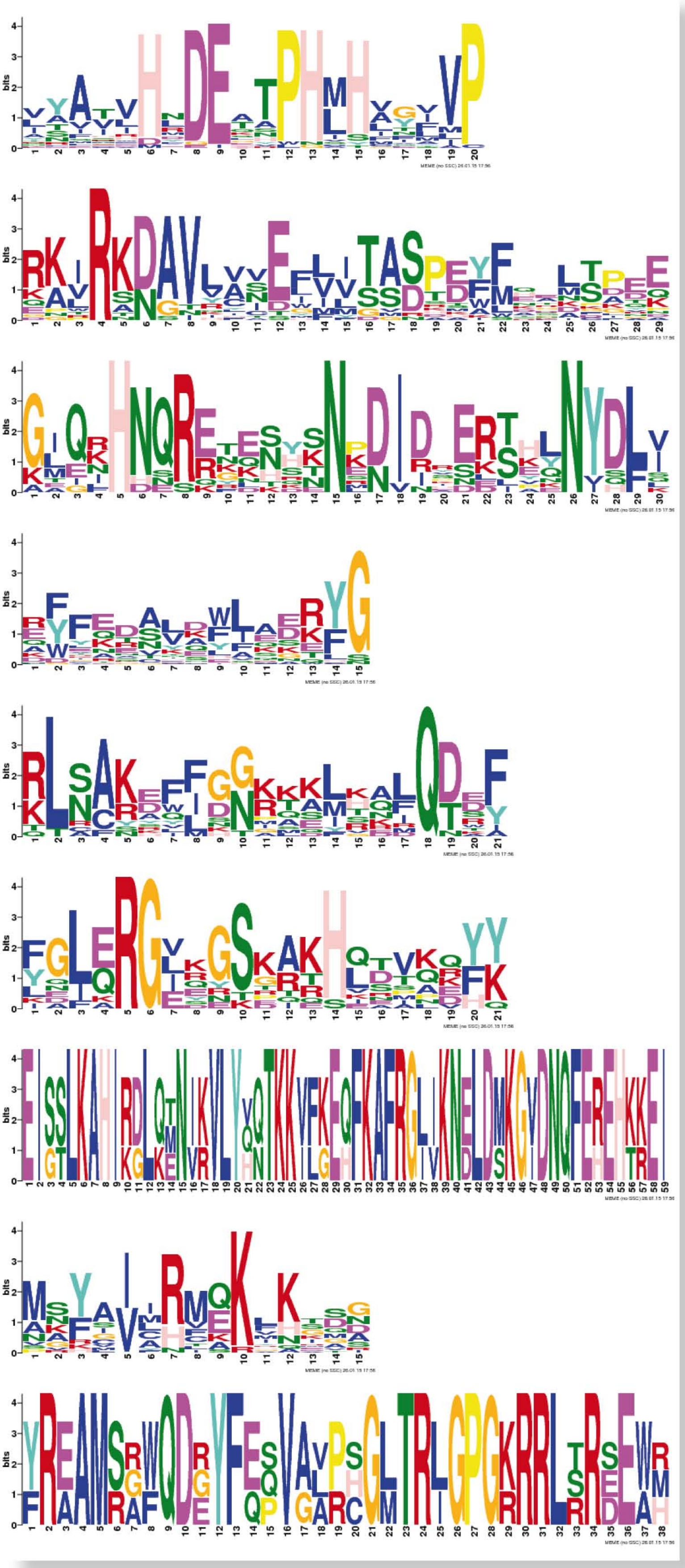

Sig	e value	Nº
1	1.4 e-240	30
2	9.6 e-122	20
3	3.1 e-83	12
4	6.7 e-57	30
5	5.7 e-35	14
6	6.4 e-32	12
7	1.7 e-28	3
8	5.4 e-26	18
9	2.6 e-17	3

D

Signatures MOB_F family

Sig		e value	Nº
1	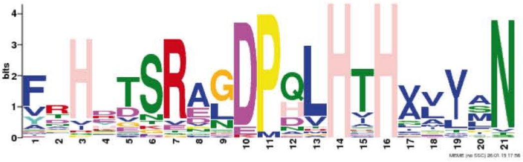	4.5 e-306	38
2	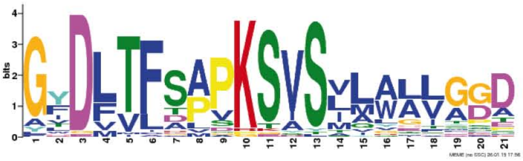	6.5 e-270	37
3	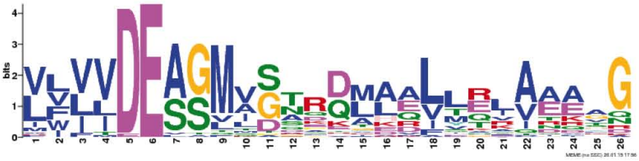	1.5 e-189	36
4	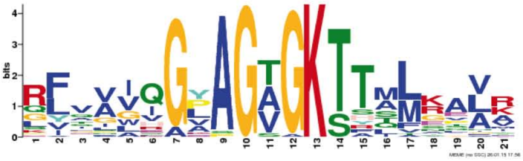	3.6 e-175	35
5	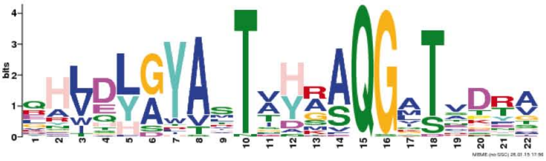	1.7 e-204	36
6	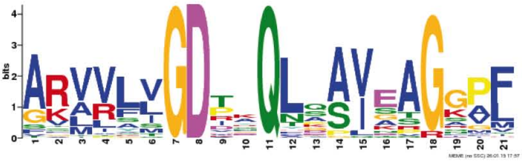	3.9 e-181	35
7	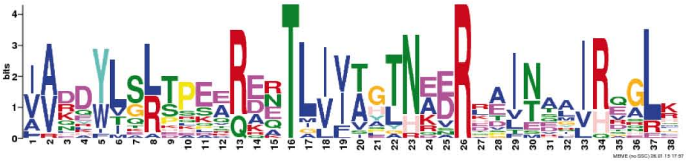	1.8 e-120	18
8	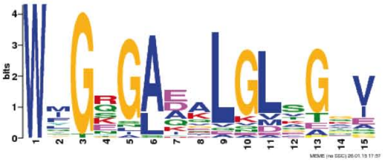	3.8 e-63	28
9	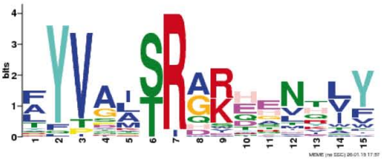	1.0 e-92	34
10	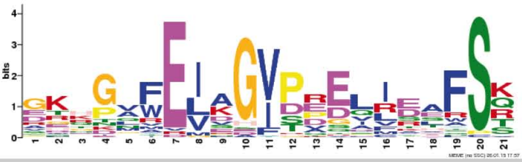	7.2 e-112	34

E

Signatures MOB_H family

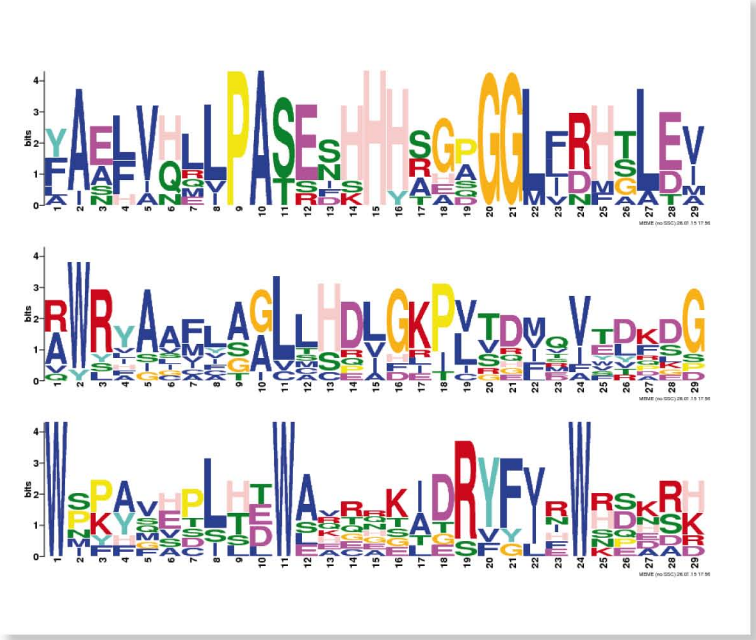

Sig	e value	Nº
1	1.5 e-58	8
2	1.5 e-32	10
3	5.4 e-25	7

F

Signatures MOB_C family

Sig		e value	Nº
1	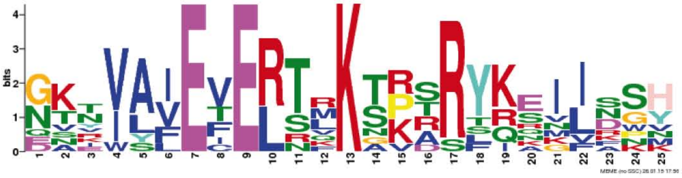	1.8 e-27	10
2	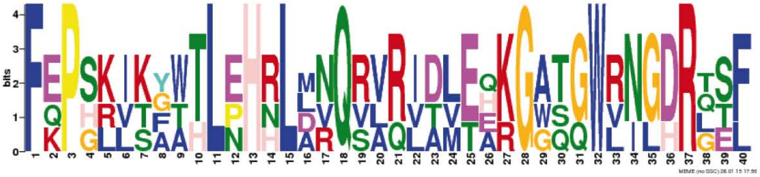	3.5 e-19	4
