# Supplementary material for: Discovery of a new family of relaxases in Firmicutes bacteria
Source: PLoS Genet. 2017 Feb 16;13(2):e1006586. doi: 10.1371/journal.pgen.1006586 (PMC5313138; doi:10.1371/journal.pgen.1006586)
Supplement: S1 Table — (PDF) [file pgen.1006586.s007.pdf]

Accesion.version	E-value	Organism	Phylum
WP_013603221.1	2E-102	Bacillus subtilis	Firmicutes
WP_033728942.1	3E-98	Bacillus weihenstephanensis	Firmicutes
WP_000093051.1	7E-98	Bacillus cereus	Firmicutes
WP_036091936.1	2E-97	Listeriaceae bacterium FSL M6-0635	Firmicutes
WP_002187836.1	3E-97	Bacillus cereus	Firmicutes
WP_002204212.1	3E-97	Bacillus cereus	Firmicutes
WP_018783109.1	3E-97	Bacillus sp. 95MFCvi2.1	Firmicutes
WP_002166614.1	4E-97	Bacillus cereus	Firmicutes
WP_000063693.1	8E-97	Bacillus thuringiensis	Firmicutes
WP_016093909.1	8E-97	Bacillus cereus	Firmicutes
WP_002151031.1	9E-97	Bacillus cereus	Firmicutes
WP_012263715.1	1E-96	Bacillus thuringiensis	Firmicutes
WP_000606311.1	2E-96	Bacillus cereus	Firmicutes
WP_016119360.1	2E-96	Bacillus cereus	Firmicutes
WP_000063692.1	2E-96	Bacillus cereus	Firmicutes
WP_033796261.1	2E-96	Bacillus mycoides	Firmicutes
WP_016105659.1	2E-96	Bacillus cereus	Firmicutes
WP_000063691.1	3E-96	Bacillus cereus	Firmicutes
WP_016513357.1	3E-96	Bacillus cereus	Firmicutes
WP_028784619.1	3E-96	Thalassobacillus devorans	Firmicutes
WP_001057731.1	4E-96	Bacillus cereus	Firmicutes
WP_008590576.1	4E-96	Salimicrobium sp. MJ3	Firmicutes
WP_000063694.1	4E-96	Bacillus cereus	Firmicutes
EEM10632.1	4E-96	Bacillus mycoides Rock3-17	Firmicutes
WP_000063690.1	5E-96	Bacillus cereus	Firmicutes
WP_000063689.1	5E-96	Bacillus cereus	Firmicutes
WP_016102511.1	5E-96	Bacillus cereus	Firmicutes
WP_035548780.1	6E-96	Halobacillus sp. BBL2006	Firmicutes
KEZ79999.1	6E-96	Bacillus weihenstephanensis	Firmicutes
WP_002191967.1	7E-96	Bacillus cereus	Firmicutes
WP_001057948.1	7E-96	Bacillus cereus	Firmicutes
WP_000063695.1	7E-96	Bacillus cereus	Firmicutes
WP_002089986.1	8E-96	Bacillus cereus	Firmicutes
WP_008637125.1	1E-95	Halobacillus sp. BAB-2008	Firmicutes
WP_003204209.1	1E-95	Bacillus mycoides	Firmicutes
WP_002193611.1	1E-95	Bacillus cereus	Firmicutes
WP_014644920.1	1E-95	Halobacillus halophilus	Firmicutes
WP_002205105.1	1E-95	Bacillus cereus	Firmicutes
WP_000063697.1	2E-95	Bacillus cereus	Firmicutes
WP_025147935.1	2E-95	Bacillus sp. H1a	Firmicutes
WP_010760170.1	3E-95	Enterococcus pallens	Firmicutes
WP_016128048.1	4E-95	Bacillus cereus	Firmicutes
WP_035507940.1	4E-95	Halobacillus	Firmicutes
WP_002130409.1	4E-95	Bacillus cereus	Firmicutes
WP_033798168.1	5E-95	Bacillus mycoides	Firmicutes
WP_001057949.1	6E-95	Bacillus cereus	Firmicutes
WP_029442968.1	7E-95	Bacillus thuringiensis	Firmicutes
WP_025966431.1	8E-95	Bacillus cereus	Firmicutes
WP_016128429.1	9E-95	Bacillus cereus	Firmicutes
WP_016372365.1	1E-94	Lactobacillus paracasei	Firmicutes
WP_002169630.1	1E-94	Bacillus cereus	Firmicutes
KFN04666.1	2E-94	Bacillus mycoides	Firmicutes
WP_018395847.1	2E-94	Bacillus sp. 37MA	Firmicutes
WP_002358243.1	2E-94	Enterococcus faecalis	Firmicutes

WP_033780699.1	2E-94	Enterococcus faecalis	Firmicutes
WP_010715713.1	3E-94	Enterococcus faecalis	Firmicutes
WP_010785148.1	3E-94	Enterococcus faecalis	Firmicutes
WP_010717251.1	3E-94	Enterococcus faecalis	Firmicutes
WP_014641918.1	4E-94	Halobacillus halophilus	Firmicutes
WP_003593122.1	4E-94	Lactobacillus casei	Firmicutes
WP_025013808.1	4E-94	Lactobacillus	Firmicutes
WP_010821328.1	4E-94	Enterococcus faecalis	Firmicutes
WP_001057732.1	4E-94	Bacillus thuringiensis	Firmicutes
WP_015543650.1	6E-94	Enterococcus sp. 7L76	Firmicutes
WP_003597600.1	6E-94	Lactobacillus casei	Firmicutes
WP_016121217.1	6E-94	Bacillus cereus	Firmicutes
WP_016383874.1	6E-94	Lactobacillus paracasei	Firmicutes
WP_010717543.1	6E-94	Enterococcus faecalis	Firmicutes
WP_016110483.1	7E-94	Bacillus cereus	Firmicutes
WP_023894696.1	7E-94	Enterococcus faecalis	Firmicutes
WP_016107045.1	7E-94	Bacillus cereus	Firmicutes
WP_016383855.1	7E-94	Lactobacillus paracasei	Firmicutes
WP_010707424.1	7E-94	Enterococcus faecalis	Firmicutes
WP_019887489.1	8E-94	Lactobacillus paracasei	Firmicutes
WP_016379706.1	8E-94	Lactobacillus	Firmicutes
WP_002358469.1	8E-94	Enterococcus faecalis	Firmicutes
WP_016370736.1	9E-94	Lactobacillus paracasei	Firmicutes
WP_016386558.1	1E-93	Lactobacillus paracasei	Firmicutes
WP_003660097.1	1E-93	Lactobacillus paracasei	Firmicutes
WP_033678232.1	1E-93	Bacillus gaemokensis	Firmicutes
WP_003570613.1	1E-93	Lactobacillus	Firmicutes
WP_016388777.1	1E-93	Lactobacillus paracasei	Firmicutes
WP_031547245.1	1E-93	Lactobacillus rhamnosus	Firmicutes
WP_016383285.1	1E-93	Lactobacillus paracasei	Firmicutes
WP_016364064.1	1E-93	Lactobacillus paracasei	Firmicutes
WP_003589994.1	2E-93	Lactobacillus casei	Firmicutes
WP_003607077.1	2E-93	Lactobacillus casei group	Firmicutes
WP_003740248.1	2E-93	Listeria monocytogenes	Firmicutes
WP_016388588.1	2E-93	Lactobacillus paracasei	Firmicutes
WP_020751452.1	2E-93	Lactobacillus casei	Firmicutes
WP_011674165.1	2E-93	Lactobacillus casei	Firmicutes
AHJ34528.1	2E-93	Lactobacillus paracasei N1115	Firmicutes
WP_003587046.1	3E-93	Lactobacillus casei	Firmicutes
WP_019897686.1	3E-93	Lactobacillus paracasei	Firmicutes
WP_000063696.1	4E-93	Bacillus cereus	Firmicutes
EPC69852.1	4E-93	Lactobacillus paracasei subsp. paracasei Lpp41	Firmicutes
WP_010529081.1	4E-93	Lentibacillus jeotgali	Firmicutes
WP_010777764.1	4E-93	Enterococcus faecalis	Firmicutes
WP_016385204.1	5E-93	Lactobacillus paracasei	Firmicutes
WP_016387292.1	5E-93	Lactobacillus paracasei	Firmicutes
WP_016381630.1	5E-93	Lactobacillus paracasei	Firmicutes
WP_019884466.1	6E-93	Lactobacillus paracasei	Firmicutes
EPD09475.1	8E-93	Lactobacillus paracasei subsp. paracasei Lpp48	Firmicutes
WP_016373125.1	1E-92	Lactobacillus paracasei	Firmicutes
WP_019891173.1	1E-92	Lactobacillus paracasei	Firmicutes
WP_016381681.1	1E-92	Lactobacillus paracasei	Firmicutes
WP_016383000.1	1E-92	Lactobacillus paracasei	Firmicutes
WP_002413217.1	1E-92	Enterococcus faecalis	Firmicutes
KGP70591.1	2E-92	Lactobacillus casei	Firmicutes

WP_003186655.1	2E-92	Bacillus licheniformis	Firmicutes
WP_016379700.1	2E-92	Lactobacillus paracasei	Firmicutes
EPC69878.1	2E-92	Lactobacillus paracasei subsp. paracasei Lpp41	Firmicutes
WP_034655615.1	2E-92	Bacillus megaterium	Firmicutes
WP_003582073.1	3E-92	Lactobacillus casei	Firmicutes
WP_031547032.1	3E-92	Lactobacillus rhamnosus	Firmicutes
WP_012491069.1	4E-92	Lactobacillus casei group	Firmicutes
WP_010529469.1	8E-92	Lentibacillus jeotgali	Firmicutes
EPC70981.1	2E-91	Lactobacillus paracasei subsp. paracasei Lpp41	Firmicutes
WP_010782477.1	2E-91	Enterococcus gilvus	Firmicutes
WP_010531158.1	4E-91	Lentibacillus jeotgali	Firmicutes
WP_003583440.1	6E-91	Lactobacillus casei	Firmicutes
WP_010493216.1	8E-91	Lactobacillus casei group	Firmicutes
WP_010711773.1	1E-90	Enterococcus faecalis	Firmicutes
WP_034656079.1	3E-90	Bacillus megaterium	Firmicutes
WP_016632474.1	4E-90	Enterococcus faecalis	Firmicutes
WP_016633139.1	4E-90	Enterococcus faecalis	Firmicutes
WP_016616630.1	8E-90	Enterococcus faecalis	Firmicutes
WP_010815379.1	1E-89	Enterococcus faecalis	Firmicutes
WP_019392513.1	1E-89	Bacillus endophyticus	Firmicutes
WP_016616489.1	2E-89	Enterococcus faecalis	Firmicutes
WP_014862505.1	4E-89	Enterococcus faecalis	Firmicutes
WP_029325353.1	4E-89	Bacillus sp. RP1137	Firmicutes
WP_010818214.1	1E-88	Enterococcus faecalis	Firmicutes
WP_002350170.1	3E-88	Enterococcus faecium	Firmicutes
WP_003290481.1	7E-88	Bacillus thuringiensis	Firmicutes
WP_029325432.1	3E-87	Bacillus sp. RP1137	Firmicutes
EEL19602.1	3E-87	Bacillus cereus Rock1-3	Firmicutes
EEL36934.1	7E-87	Bacillus cereus Rock3-29	Firmicutes
WP_031647240.1	8E-87	Listeria monocytogenes	Firmicutes
WP_003203645.1	1E-85	Bacillus mycoides	Firmicutes
WP_031659845.1	1E-85	Listeria monocytogenes	Firmicutes
WP_038286203.1	2E-85	[Clostridium] bifermentans	Firmicutes
WP_003310141.1	2E-85	Bacillus thuringiensis	Firmicutes
WP_004233231.1	6E-85	Lysinibacillus fusiformis	Firmicutes
WP_003725254.1	2E-84	Listeria monocytogenes	Firmicutes
WP_016366324.1	2E-84	Lactobacillus paracasei	Firmicutes
BAN72694.1	3E-84	Lactobacillus paracasei subsp. paracasei JCM 8130	Firmicutes
WP_036124811.1	5E-83	Lysinibacillus	Firmicutes
WP_014386599.1	6E-83	Lactococcus garvieae	Firmicutes
WP_017371065.1	2E-82	Lactococcus garvieae	Firmicutes
WP_036165803.1	1E-81	Lysinibacillus sphaericus	Firmicutes
EUJ42869.1	2E-81	Listeria riparia FSL S10-1204	Firmicutes
WP_001095810.1	3E-81	Bacillus cereus	Firmicutes
WP_033694044.1	3E-81	Bacillus cereus	Firmicutes
CDQ37255.1	6E-80	Virgibacillus halodenitrificans	Firmicutes
WP_031417611.1	7E-80	Lysinibacillus sphaericus	Firmicutes
WP_001095808.1	9E-80	Bacillus cereus	Firmicutes
WP_034808439.1	1E-79	Exiguobacterium sp. AB2	Firmicutes
WP_001095809.1	2E-79	Bacillus thuringiensis	Firmicutes
WP_026824333.1	1E-78	Exiguobacterium marinum	Firmicutes
WP_031424837.1	2E-78	Exiguobacterium sp. NG55	Firmicutes
WP_024372255.1	2E-78	Exiguobacterium sp. 8-11-1	Firmicutes
WP_012390438.1	5E-78	Exiguobacterium arabatum	Firmicutes
WP_021065848.1	1E-77	Exiguobacterium pavilionensis	Firmicutes

WP_003136439.1	1E-77	Lactococcus garvieae	Firmicutes
WP_029596486.1	2E-77	Exiguobacterium sp. GIC31	Firmicutes
WP_016382362.1	5E-77	Lactobacillus paracasei	Firmicutes
WP_024127780.1	7E-77	Exiguobacterium sp. S3-2	Firmicutes
WP_031424731.1	9E-77	Exiguobacterium sp. NG55	Firmicutes
CEJ75502.1	2E-76	[Clostridium] sordellii	Firmicutes
WP_025809625.1	2E-76	Bacillus licheniformis	Firmicutes
WP_036142074.1	2E-76	Lysinibacillus sp. BF-4	Firmicutes
CEK32652.1	3E-76	[Clostridium] sordellii	Firmicutes
CEK36627.1	3E-76	[Clostridium] sordellii	Firmicutes
WP_021430704.1	3E-76	[Clostridium] bifermentans	Firmicutes
WP_035399428.1	4E-76	Exiguobacterium sp. OS-77	Firmicutes
WP_032859152.1	4E-76	Bacillus amyloliquefaciens	Firmicutes
WP_029336116.1	5E-76	Exiguobacterium aurantiacum	Firmicutes
AIW54804.1	8E-76	Clostridium botulinum	Firmicutes
WP_017370757.1	1E-75	Lactococcus garvieae	Firmicutes
WP_021127617.1	2E-75	[Clostridium] sordellii	Firmicutes
WP_012431069.1	3E-75	Clostridium botulinum	Firmicutes
WP_016362211.1	4E-75	Bacillus cereus	Firmicutes
WP_035784585.1	5E-75	Clostridium botulinum	Firmicutes
WP_003474246.1	8E-75	Clostridium perfringens	Firmicutes
WP_021424744.1	2E-74	Peptoclostridium difficile	Firmicutes
WP_025370664.1	3E-74	Listeria monocytogenes	Firmicutes
WP_011011014.1	3E-74	Listeria	Firmicutes
WP_003728490.1	3E-74	Listeria	Firmicutes
WP_031645643.1	4E-74	Listeria monocytogenes	Firmicutes
WP_011461991.1	4E-74	Desulfitobacterium hafniense	Firmicutes
ACJ66900.1	5E-74	Bacillus subtilis	Firmicutes
WP_031670160.1	9E-74	Listeria monocytogenes	Firmicutes
WP_031641553.1	9E-74	Listeria monocytogenes	Firmicutes
WP_031642432.1	1E-73	Listeria	Firmicutes
WP_035412834.1	1E-73	Exiguobacterium sp. RIT341	Firmicutes
WP_011264114.1	1E-73	Clostridium perfringens	Firmicutes
WP_003464984.1	1E-73	Clostridium perfringens	Firmicutes
WP_012281334.1	1E-73	Heliobacterium modesticaldum	Firmicutes
WP_010968268.1	1E-73	Clostridium perfringens	Firmicutes
WP_002396563.1	1E-73	Enterococcus faecalis	Firmicutes
WP_003482620.1	2E-73	Clostridium perfringens	Firmicutes
WP_021315449.1	2E-73	Dehalobacter sp. UNSWDHB	Firmicutes
WP_003453260.1	2E-73	Clostridium perfringens	Firmicutes
WP_004461164.1	2E-73	Clostridium perfringens	Firmicutes
WP_034865965.1	4E-73	Enterococcus faecium	Firmicutes
WP_024269847.1	6E-73	Clostridium perfringens	Firmicutes
EOJ91113.1	7E-73	Enterococcus faecalis EnGen0368	Firmicutes
WP_016368668.1	7E-73	Lactobacillus paracasei	Firmicutes
WP_003464757.1	1E-72	Clostridium perfringens	Firmicutes
WP_024345857.1	1E-72	[Clostridium] methoxybenzovorans	Firmicutes
WP_015043417.1	3E-72	Dehalobacter sp. DCA	Firmicutes
WP_033120500.1	4E-72	Oscillibacter sp. ER4	Firmicutes
WP_023558802.1	4E-72	Listeria monocytogenes	Firmicutes
WP_003458987.1	5E-72	Clostridium perfringens	Firmicutes
WP_033165265.1	8E-72	Clostridium sp. KNHs205	Firmicutes
WP_024345917.1	1E-71	[Clostridium] methoxybenzovorans	Firmicutes
WP_009261519.1	3E-71	Lachnospiraceae bacterium 7_1_58FAA	Firmicutes
WP_024041493.1	7E-71	Enterococcus faecalis	Firmicutes

WP_014314916.1	1E-70	Clostridium sp. BNL1100	Firmicutes
WP_021631428.1	1E-70	Clostridium sp. ATCC BAA-442	Firmicutes
WP_003480913.1	1E-70	Clostridium perfringens	Firmicutes
WP_016615245.1	2E-70	Enterococcus faecalis	Firmicutes
WP_033919037.1	2E-70	Enterococcus faecalis	Firmicutes
WP_007493928.1	2E-70	Flavonifractor plautii	Firmicutes
WP_029343419.1	2E-70	Exiguobacterium acetylicum	Firmicutes
WP_009261383.1	2E-70	Lachnospiraceae bacterium 7_1_58FAA	Firmicutes
WP_035387538.1	3E-70	Exiguobacterium undae	Firmicutes
WP_025543755.1	3E-70	Oscillospiraceae bacterium VE202-24	Firmicutes
WP_002360837.1	3E-70	Enterococcus faecalis	Firmicutes
WP_006876450.1	3E-70	Clostridiales	Firmicutes
WP_007492818.1	6E-70	Flavonifractor plautii	Firmicutes
WP_035413953.1	7E-70	Exiguobacterium undae	Firmicutes
WP_014862330.1	1E-69	Enterococcus faecalis	Firmicutes
WP_000520462.1	1E-69	Bacillus cereus	Firmicutes
WP_002203814.1	1E-69	Bacillus cereus	Firmicutes
WP_015043306.1	2E-69	Dehalobacter	Firmicutes
WP_016085092.1	2E-69	Bacillus cereus	Firmicutes
WP_015262211.1	3E-69	Desulfitobacterium dichloroeliminans	Firmicutes
WP_018306610.1	3E-69	Desulfitobacterium hafniense	Firmicutes
WP_009261422.1	3E-69	Lachnospiraceae bacterium 7_1_58FAA	Firmicutes
CEK36536.1	4E-69	[Clostridium] sordellii	Firmicutes
WP_011461899.1	5E-69	Desulfitobacterium hafniense	Firmicutes
WP_010730140.1	5E-69	Enterococcus faecalis	Firmicutes
WP_003476238.1	6E-69	Clostridium perfringens	Firmicutes
WP_006520256.1	6E-69	Desulfotomaculum gibsoniae	Firmicutes
WP_009269001.1	6E-69	Lachnospiraceae bacterium 1_4_56FAA	Firmicutes
WP_000520467.1	7E-69	Bacillus cereus	Firmicutes
WP_016622191.1	8E-69	Enterococcus faecalis	Firmicutes
WP_009261387.1	9E-69	Lachnospiraceae bacterium 7_1_58FAA	Firmicutes
WP_015261860.1	1E-68	Desulfitobacterium dichloroeliminans	Firmicutes
WP_014120029.1	2E-68	Oscillibacter valericigenes	Firmicutes
WP_016320789.1	2E-68	Oscillibacter sp. 1-3	Firmicutes
ELA94325.1	2E-68	Enterococcus faecium EnGen0018	Firmicutes
WP_034566125.1	3E-68	Clostridiales bacterium VE202-03	Firmicutes
WP_015049874.1	3E-68	Thermacetogenium phaeum	Firmicutes
WP_028308204.1	5E-68	Desulfitibacter alkalitolerans	Firmicutes
KGF54460.1	5E-68	Clostridium orbiscindens 1_3_50AFAA	Firmicutes
WP_033117674.1	6E-68	Intestinimonas butyriciproducens	Firmicutes
WP_009261480.1	6E-68	Lachnospiraceae bacterium 7_1_58FAA	Firmicutes
WP_016099589.1	7E-68	Bacillus cereus	Firmicutes
EOF56271.1	8E-68	Enterococcus hirae EnGen0127	Firmicutes
WP_014314988.1	8E-68	Clostridium sp. BNL1100	Firmicutes
WP_010824079.1	9E-68	Enterococcus faecalis	Firmicutes
WP_008381272.1	1E-67	Enterococcus sp. C1	Firmicutes
WP_034421000.1	1E-67	Clostridiales bacterium DRI-13	Firmicutes
WP_013623282.1	1E-67	Syntrophobotulus glycolicus	Firmicutes
WP_027096097.1	1E-67	[Clostridium] viride	Firmicutes
WP_000520466.1	2E-67	Bacillus cereus	Firmicutes
KGF52123.1	2E-67	Clostridium orbiscindens 1_3_50AFAA	Firmicutes
WP_004612173.1	2E-67	Tyzzerella nexilis	Firmicutes
ERI89931.1	2E-67	Clostridiales bacterium oral taxon 876 str. F0540	Firmicutes
WP_026889881.1	2E-67	[Clostridium] aerotolerans	Firmicutes
WP_006876431.1	2E-67	Anaerotruncus colihominis	Firmicutes

WP_016078860.1	3E-67	Bacillus cereus	Firmicutes
WP_016128084.1	3E-67	Bacillus cereus	Firmicutes
WP_023348694.1	4E-67	Firmicutes bacterium ASF500	Firmicutes
WP_009257249.1	5E-67	Lachnospiraceae bacterium 7_1_58FAA	Firmicutes
WP_009261489.1	5E-67	Lachnospiraceae bacterium 7_1_58FAA	Firmicutes
WP_015757089.1	5E-67	Desulfotomaculum acetoxidans	Firmicutes
WP_009261527.1	6E-67	Lachnospiraceae bacterium 7_1_58FAA	Firmicutes
WP_034567356.1	6E-67	Clostridiales bacterium VE202-03	Firmicutes
WP_016097311.1	7E-67	Bacillus cereus group	Firmicutes
WP_018213967.1	7E-67	Desulfitobacterium hafniense	Firmicutes
WP_018307450.1	1E-66	Desulfitobacterium hafniense	Firmicutes
WP_016610085.1	1E-66	Enterococcus casseliflavus	Firmicutes
WP_024731083.1	2E-66	Clostridiales bacterium VE202-13	Firmicutes
WP_000520464.1	2E-66	Bacillus cereus	Firmicutes
WP_024723266.1	3E-66	Clostridiales bacterium VE202-03	Firmicutes
WP_020989589.1	3E-66	Ruminococcaceae bacterium D16	Firmicutes
WP_016612466.1	3E-66	Enterococcus faecium	Firmicutes
WP_014120230.1	3E-66	Oscillibacter valericigenes	Firmicutes
EOH52452.1	3E-66	Enterococcus faecium EnGen0263	Firmicutes
WP_021749214.1	3E-66	Oscillibacter sp. KLE 1728	Firmicutes
EUC51766.1	3E-66	Mogibacterium timidum ATCC 33093	Firmicutes
WP_006877028.1	5E-66	Anaerotruncus colihominis	Firmicutes
WP_011458828.1	7E-66	Desulfitobacterium hafniense	Firmicutes
CDW99993.1	8E-66	Desulfitobacterium hafniense	Firmicutes
WP_020072859.1	1E-65	[Clostridium] sporosphaeroides	Firmicutes
EOK08875.1	1E-65	Enterococcus faecalis ATCC 6055	Firmicutes
WP_014990432.1	1E-65	Bacillus thuringiensis	Firmicutes
WP_000520468.1	2E-65	Bacillus cereus	Firmicutes
WP_036629333.1	2E-65	Oscillibacter	Firmicutes
EGV02919.1	2E-65	Streptococcus infantis SK970	Firmicutes
ERK63996.1	4E-65	Oscillibacter sp. KLE 1728	Firmicutes
WP_002198667.1	5E-65	Bacillus cereus	Firmicutes
WP_023383192.1	6E-65	Youngiibacter fragilis	Firmicutes
WP_025543361.1	8E-65	Oscillospiraceae bacterium VE202-24	Firmicutes
WP_018377910.1	1E-64	Streptococcus ovis	Firmicutes
WP_016323200.1	1E-64	Oscillibacter sp. 1-3	Firmicutes
WP_022795075.1	2E-64	Marinococcus halotolerans	Firmicutes
WP_002321294.1	2E-64	Enterococcus faecium	Firmicutes
WP_013273751.1	2E-64	[Clostridium] saccharolyticum	Firmicutes
WP_022166040.1	2E-64	Eubacterium sp. CAG:192	Firmicutes
WP_023043048.1	3E-64	Enterococcus faecium	Firmicutes
WP_033705138.1	4E-64	Enterococcus faecium	Firmicutes
WP_002261841.1	4E-64	Streptococcus mutans	Firmicutes
WP_016619380.1	6E-64	Enterococcus faecalis	Firmicutes
WP_000520460.1	6E-64	Bacillus thuringiensis	Firmicutes
WP_000520463.1	6E-64	Bacillus anthracis	Firmicutes
WP_002337950.1	8E-64	Enterococcus faecium	Firmicutes
WP_009257220.1	1E-63	Clostridiales	Firmicutes
WP_016084437.1	1E-63	Bacillus cereus	Firmicutes
WP_017650553.1	1E-63	Streptococcus agalactiae	Firmicutes
WP_018375463.1	1E-63	Streptococcus orisratti	Firmicutes
WP_010729518.1	1E-63	Enterococcus faecium	Firmicutes
WP_014116791.1	1E-63	Oscillibacter valericigenes	Firmicutes
WP_035349209.1	2E-63	Bacillus ginsengihumi	Firmicutes
WP_025600795.1	2E-63	Lactobacillus casei	Firmicutes

WP_002588938.1	2E-63	[Clostridium] clostridioforme	Firmicutes
WP_023523815.1	2E-63	Enterococcus mundtii	Firmicutes
WP_033607136.1	2E-63	Enterococcus faecium	Firmicutes
WP_017646904.1	2E-63	Streptococcus agalactiae	Firmicutes
WP_002320573.1	2E-63	Enterococcus faecium	Firmicutes
WP_002361217.1	2E-63	Enterococcus faecium	Firmicutes
EOS22325.1	2E-63	Lachnospiraceae bacterium A4	Firmicutes
WP_000520459.1	2E-63	Bacillus cereus	Firmicutes
WP_003572544.1	2E-63	Lactobacillus casei	Firmicutes
EPV22510.1	2E-63	Streptococcus agalactiae GB00640	Firmicutes
WP_021747541.1	2E-63	Oscillibacter	Firmicutes
ELB05607.1	3E-63	Enterococcus faecium EnGen0029	Firmicutes
WP_002587149.1	3E-63	Clostridiales	Firmicutes
WP_000520454.1	3E-63	Bacillus cereus	Firmicutes
WP_002341543.1	3E-63	Enterococcus faecium	Firmicutes
WP_010778259.1	3E-63	Enterococcus faecium	Firmicutes
WP_002351487.1	3E-63	Streptococcus mutans	Firmicutes
WP_008980802.1	3E-63	Ruminococcaceae bacterium D16	Firmicutes
WP_000520457.1	4E-63	Bacillus cereus	Firmicutes
EOH51283.1	4E-63	Enterococcus faecium EnGen0263	Firmicutes
WP_002310990.1	4E-63	Enterococcus faecium	Firmicutes
WP_002307530.1	4E-63	Enterococcus faecium	Firmicutes
EPT73189.1	4E-63	Streptococcus agalactiae CCUG 39096 A	Firmicutes
WP_000520458.1	4E-63	Bacillus cereus	Firmicutes
WP_027970844.1	4E-63	Streptococcus castoreus	Firmicutes
WP_002571517.1	4E-63	[Clostridium] bolteae	Firmicutes
WP_002330591.1	5E-63	Enterococcus faecium	Firmicutes
AII40743.1	5E-63	Enterococcus faecium T110	Firmicutes
WP_008265084.1	5E-63	Enterococcus	Firmicutes
WP_002332867.1	5E-63	Enterococcus faecium	Firmicutes
WP_033606696.1	5E-63	Enterococcus faecium	Firmicutes
WP_006857943.1	6E-63	Roseburia intestinalis	Firmicutes
WP_002296379.1	6E-63	Enterococcus faecium	Firmicutes
WP_017648516.1	6E-63	Streptococcus agalactiae	Firmicutes
WP_027641814.1	6E-63	[Clostridium] clostridioforme	Firmicutes
WP_002595383.1	6E-63	[Clostridium] clostridioforme	Firmicutes
ELA64023.1	6E-63	Enterococcus faecium EnGen0019	Firmicutes
WP_017771196.1	7E-63	Streptococcus	Firmicutes
WP_016323154.1	7E-63	Oscillibacter sp. 1-3	Firmicutes
WP_033624447.1	7E-63	Enterococcus faecium	Firmicutes
WP_005877015.1	7E-63	Enterococcus durans	Firmicutes
WP_027973726.1	7E-63	Streptococcus porci	Firmicutes
WP_037618994.1	8E-63	Streptococcus sp. AC15	Firmicutes
WP_029996592.1	8E-63	Streptococcus suis	Firmicutes
WP_024389801.1	9E-63	Streptococcus suis	Firmicutes
WP_000059461.1	9E-63	Streptococcus agalactiae	Firmicutes
WP_002363611.1	9E-63	Enterococcus faecalis	Firmicutes
WP_017650632.1	9E-63	Streptococcus agalactiae	Firmicutes
WP_007492635.1	9E-63	Flavonifractor plautii	Firmicutes
WP_002369665.1	1E-62	Enterococcus faecium	Firmicutes
WP_024417941.1	1E-62	Streptococcus suis	Firmicutes
WP_039141258.1	1E-62	Lactobacillus rhamnosus	Firmicutes
WP_034684892.1	1E-62	Enterococcus phoeniculicola	Firmicutes
WP_002334592.1	1E-62	Enterococcus	Firmicutes
WP_002573760.1	2E-62	[Clostridium] bolteae	Firmicutes

WP_010738678.1	2E-62	Enterococcus faecium	Firmicutes
WP_000383377.1	2E-62	Streptococcus agalactiae	Firmicutes
ELA88452.1	2E-62	Enterococcus faecium EnGen0009	Firmicutes
WP_014118900.1	2E-62	Oscillibacter valericigenes	Firmicutes
WP_034867276.1	2E-62	Clostridium paraputrificum	Firmicutes
WP_016324040.1	2E-62	Oscillibacter sp. 1-3	Firmicutes
EEI61636.1	2E-62	Enterococcus faecium TX1330	Firmicutes
WP_016922213.1	2E-62	Enterococcus faecium	Firmicutes
WP_002588006.1	2E-62	[Clostridium] clostridioforme	Firmicutes
EOG08007.1	2E-62	Enterococcus faecium EnGen0176	Firmicutes
EOL43000.1	3E-62	Enterococcus phoeniculicola ATCC BAA-412	Firmicutes
WP_002571398.1	3E-62	[Clostridium] bolteae	Firmicutes
WP_000383376.1	3E-62	Streptococcus agalactiae	Firmicutes
EOI38537.1	3E-62	Enterococcus faecium EnGen0313	Firmicutes
WP_000383375.1	3E-62	Streptococcus agalactiae	Firmicutes
WP_033625003.1	3E-62	Enterococcus faecium	Firmicutes
WP_002326880.1	4E-62	Enterococcus faecium	Firmicutes
WP_000520455.1	4E-62	Bacillus thuringiensis	Firmicutes
WP_029177417.1	4E-62	Streptococcus suis	Firmicutes
WP_031919411.1	4E-62	Staphylococcus aureus	Firmicutes
WP_033586138.1	4E-62	Enterococcus faecium	Firmicutes
WP_031905999.1	4E-62	Staphylococcus aureus	Firmicutes
WP_002565951.1	4E-62	[Clostridium] bolteae	Firmicutes
WP_029175518.1	5E-62	Streptococcus suis	Firmicutes
WP_001996350.1	5E-62	Bacillus cereus	Firmicutes
WP_016317181.1	5E-62	Anaerotruncus sp. G3(2012)	Firmicutes
WP_033793973.1	5E-62	Enterococcus faecium	Firmicutes
WP_010891448.1	5E-62	Bacillus anthracis	Firmicutes
WP_003605668.1	6E-62	Lactobacillus casei	Firmicutes
WP_033611853.1	6E-62	Enterococcus faecium	Firmicutes
WP_000083590.1	6E-62	Streptococcus agalactiae	Firmicutes
WP_002343429.1	6E-62	Enterococcus faecium	Firmicutes
WP_002316068.1	6E-62	Enterococcus faecium	Firmicutes
WP_001103205.1	6E-62	Streptococcus agalactiae	Firmicutes
WP_017650045.1	6E-62	Streptococcus agalactiae	Firmicutes
EHS76054.1	7E-62	Staphylococcus aureus subsp. aureus IS-189	Firmicutes
WP_002335353.1	7E-62	Enterococcus faecium	Firmicutes
WP_008400373.1	7E-62	Clostridium sp. L2-50	Firmicutes
WP_017643215.1	7E-62	Streptococcus agalactiae	Firmicutes
WP_012816501.1	8E-62	Staphylococcus aureus	Firmicutes
WP_001631996.1	8E-62	Staphylococcus aureus	Firmicutes
WP_033627878.1	8E-62	Enterococcus faecium	Firmicutes
WP_037616762.1	9E-62	Streptococcus sinensis	Firmicutes
WP_002334030.1	1E-61	Enterococcus faecium	Firmicutes
WP_033606621.1	1E-61	Enterococcus faecium	Firmicutes
EVF57953.1	1E-61	Staphylococcus aureus LPIH6011	Firmicutes
WP_017643394.1	1E-61	Streptococcus agalactiae	Firmicutes
WP_002338416.1	1E-61	Enterococcus faecium	Firmicutes
WP_023348371.1	1E-61	Firmicutes bacterium ASF500	Firmicutes
WP_033607413.1	1E-61	Enterococcus faecium	Firmicutes
EJX89385.1	1E-61	Enterococcus faecium ERV38	Firmicutes
WP_001124489.1	1E-61	Staphylococcus aureus	Firmicutes
WP_002578177.1	1E-61	Lachnoclostridium	Firmicutes
WP_031867301.1	2E-61	Staphylococcus aureus	Firmicutes
WP_033706160.1	2E-61	Enterococcus faecium	Firmicutes

WP_002341613.1	2E-61	Enterococcus faecium	Firmicutes
WP_033582318.1	2E-61	Enterococcus faecium	Firmicutes
WP_031902827.1	2E-61	Staphylococcus aureus	Firmicutes
EVF23266.1	2E-61	Staphylococcus aureus KINW6003	Firmicutes
WP_000383373.1	2E-61	Streptococcus agalactiae	Firmicutes
WP_003045920.1	2E-61	Streptococcus canis	Firmicutes
ERK64627.1	2E-61	Oscillibacter sp. KLE 1728	Firmicutes
ELA57454.1	2E-61	Enterococcus faecium EnGen0022	Firmicutes
WP_008397067.1	2E-61	Clostridium sp. M62/1	Firmicutes
WP_002325545.1	2E-61	Enterococcus faecium	Firmicutes
WP_014748764.1	2E-61	Enterococcus faecium	Firmicutes
WP_000383374.1	3E-61	Streptococcus	Firmicutes
WP_003045446.1	3E-61	Streptococcus canis	Firmicutes
WP_000520456.1	3E-61	Bacillus thuringiensis	Firmicutes
WP_001057502.1	3E-61	Streptococcus agalactiae	Firmicutes
WP_012816541.1	4E-61	Staphylococcus aureus	Firmicutes
WP_017768569.1	5E-61	Streptococcus agalactiae	Firmicutes
ELB74775.1	8E-61	Enterococcus faecium EnGen0057	Firmicutes
WP_038809477.1	9E-61	Enterococcus faecium	Firmicutes
WP_017643739.1	9E-61	Streptococcus agalactiae	Firmicutes
WP_031864843.1	1E-60	Staphylococcus aureus	Firmicutes
WP_012104176.1	1E-60	Clostridium kluyveri	Firmicutes
WP_002593792.1	1E-60	[Clostridium] clostridioforme	Firmicutes
WP_002107419.1	1E-60	Bacillus cereus	Firmicutes
WP_017643454.1	1E-60	Streptococcus agalactiae	Firmicutes
EHM32226.1	1E-60	Enterococcus faecium E4453	Firmicutes
WP_015517144.1	2E-60	Eubacterium rectale	Firmicutes
WP_016182791.1	2E-60	Enterococcus columbae	Firmicutes
WP_033655399.1	2E-60	Enterococcus faecium	Firmicutes
WP_002352778.1	3E-60	Enterococcus faecium	Firmicutes
WP_013273431.1	4E-60	[Clostridium] saccharolyticum	Firmicutes
EHE05558.1	4E-60	Streptococcus pneumoniae GA17328	Firmicutes
WP_019774100.1	5E-60	Streptococcus sobrinus	Firmicutes
WP_019793066.1	5E-60	Streptococcus sobrinus	Firmicutes
WP_004622477.1	5E-60	[Clostridium] papyrosolvens	Firmicutes
WP_012951712.1	6E-60	Listeria monocytogenes	Firmicutes
WP_027829215.1	8E-60	Lactobacillus harbinensis	Firmicutes
WP_019792357.1	8E-60	Streptococcus sobrinus	Firmicutes
WP_019777173.1	8E-60	Streptococcus sobrinus	Firmicutes
CDQ37721.1	1E-59	Virgibacillus halodenitrificans	Firmicutes
WP_019781702.1	1E-59	Streptococcus sobrinus	Firmicutes
WP_015520840.1	1E-59	Roseburia intestinalis	Firmicutes
EOM66387.1	1E-59	Enterococcus faecium EnGen0165	Firmicutes
WP_012655854.1	2E-59	Macrococcus caseolyticus	Firmicutes
WP_003476279.1	2E-59	Clostridium perfringens	Firmicutes
WP_009730741.1	2E-59	Streptococcus sp. F0441	Firmicutes
WP_020006627.1	2E-59	Salinicoccus albus	Firmicutes
WP_006569668.1	2E-59	Thermoanaerobacter siderophilus	Firmicutes
WP_031770318.1	3E-59	Staphylococcus aureus	Firmicutes
WP_017648560.1	4E-59	Streptococcus agalactiae	Firmicutes
WP_016324475.1	4E-59	Oscillibacter sp. 1-3	Firmicutes
WP_019907142.1	4E-59	Thermoanaerobacter indiensis	Firmicutes
AHZ48436.1	5E-59	Streptococcus sp. VT 162	Firmicutes
WP_029237954.1	6E-59	Streptococcus sobrinus	Firmicutes
WP_016119554.1	6E-59	Bacillus cereus	Firmicutes

WP_002367862.1	7E-59	Enterococcus faecium	Firmicutes
EIC78810.1	8E-59	Streptococcus oralis SK10	Firmicutes
KEQ49638.1	9E-59	Streptococcus oralis	Firmicutes
WP_013570759.1	1E-58	Thermoanaerobacter brockii	Firmicutes
WP_021915705.1	1E-58	Firmicutes bacterium CAG:24	Firmicutes
EGV14959.1	1E-58	Streptococcus infantis X	Firmicutes
WP_003476060.1	2E-58	Clostridium perfringens	Firmicutes
WP_001103206.1	2E-58	Streptococcus sp. oral taxon 071	Firmicutes
WP_033584653.1	3E-58	Streptococcus sp. SR1	Firmicutes
WP_001103207.1	4E-58	Streptococcus agalactiae	Firmicutes
WP_003457695.1	4E-58	Clostridium perfringens	Firmicutes
WP_022416698.1	6E-58	Eubacterium sp. CAG:841	Firmicutes
WP_002352206.1	1E-57	Enterococcus faecium	Firmicutes
WP_007520050.1	1E-57	Streptococcus tigurinus	Firmicutes
WP_002349329.1	1E-57	Enterococcus faecium	Firmicutes
WP_001574993.1	1E-57	Staphylococcus aureus	Firmicutes
WP_039671377.1	2E-57	Streptococcus macedonicus	Firmicutes
WP_019789872.1	2E-57	Streptococcus sobrinus	Firmicutes
WP_020916760.1	2E-57	Streptococcus lutetiensis	Firmicutes
WP_039695706.1	2E-57	Streptococcus gallolyticus	Firmicutes
WP_015521360.1	3E-57	Roseburia intestinalis	Firmicutes
WP_003461650.1	3E-57	Clostridium perfringens	Firmicutes
WP_009609813.1	3E-57	Caldanaerobacter subterraneus	Firmicutes
WP_014620289.1	7E-57	Streptococcus gallolyticus	Firmicutes
WP_033616817.1	8E-57	Enterococcus faecium	Firmicutes
EJX75017.1	9E-57	Enterococcus faecium P1123	Firmicutes
WP_023349023.1	4E-56	Firmicutes bacterium ASF500	Firmicutes
WP_013149542.1	5E-56	Thermoanaerobacter mathranii	Firmicutes
WP_002487745.1	6E-56	Staphylococcus epidermidis	Firmicutes
WP_024725799.1	2E-55	Clostridiales bacterium VE202-07	Firmicutes
WP_036130006.1	2E-55	Listeriaceae bacterium FSL A5-0209	Firmicutes
WP_006739082.1	2E-55	Streptococcus urinalis	Firmicutes
KGK75347.1	3E-55	Enterococcus faecium	Firmicutes
WP_002349290.1	5E-55	Enterococcus faecium	Firmicutes
WP_016083864.1	8E-55	Bacillus cereus	Firmicutes
WP_007884334.1	1E-54	Roseburia inulinivorans	Firmicutes
WP_021970481.1	1E-54	Bacteroides sp. CAG:1076	Bacteroidetes
EEU69504.1	3E-54	Enterococcus faecalis Merz96	Firmicutes
WP_008809727.1	4E-54	Streptococcus sp. 2_1_36FAA	Firmicutes
WP_032773815.1	4E-54	Lactobacillus paracasei	Firmicutes
WP_004615144.1	5E-54	Tyzzerella nexilis	Firmicutes
WP_002587162.1	5E-54	Clostridiales	Firmicutes
WP_013245284.1	5E-54	Bacillus cereus	Firmicutes
WP_037582762.1	8E-54	Streptococcus anginosus	Firmicutes
WP_021681546.1	2E-53	Ruminococcus callidus	Firmicutes
WP_004615549.1	2E-53	Tyzzerella nexilis	Firmicutes
WP_037612620.1	3E-53	Streptococcus sp. OBRC6	Firmicutes
ETI85220.1	3E-53	Streptococcus anginosus DORA_7	Firmicutes
WP_021949985.1	4E-53	Clostridium sp. CAG:678	Firmicutes
WP_021932381.1	9E-53	Firmicutes bacterium CAG:124	Firmicutes
WP_003078061.1	1E-52	Streptococcus intermedius	Firmicutes
WP_010749197.1	1E-52	Enterococcus casseliflavus	Firmicutes
WP_012387005.1	1E-52	Bacillus thuringiensis	Firmicutes
BAN61410.1	1E-52	Streptococcus anginosus subsp. whileyi MAS624	Firmicutes
WP_023916851.1	3E-52	Streptococcus sanguinis	Firmicutes

WP_005237636.1	4E-52	Enterococcus casseliflavus	Firmicutes
WP_009322745.1	4E-52	Subdoligranulum sp. 4_3_54A2FAA	Firmicutes
WP_015518983.1	2E-51	Clostridiales	Firmicutes
WP_005230846.1	2E-51	Enterococcus casseliflavus	Firmicutes
WP_013296849.1	3E-51	Thermoanaerobacterium thermosaccharolyticum	Firmicutes
WP_037329073.1	4E-51	Ruminococcus flavefaciens	Firmicutes
WP_015566966.1	9E-51	[Eubacterium] siraeum	Firmicutes
WP_034631213.1	2E-50	Desulfotomaculum alcoholivorax	Firmicutes
WP_015566801.1	3E-50	[Eubacterium] siraeum	Firmicutes
WP_015518510.1	5E-50	[Eubacterium] siraeum	Firmicutes
WP_012087613.1	5E-50	Staphylococcus epidermidis	Firmicutes
EVT89266.1	6E-50	Bacillus anthracis 8903-G	Firmicutes
WP_035066868.1	7E-50	Carnobacterium maltaromaticum	Firmicutes
WP_035425173.1	8E-50	Lactobacillus gasseri	Firmicutes
WP_022145610.1	8E-50	Firmicutes bacterium CAG:238	Firmicutes
WP_006588351.1	1E-49	Lactobacillus jensenii	Firmicutes
WP_033608209.1	1E-49	Lactobacillus plantarum	Firmicutes
WP_000513654.1	2E-49	Bacillus anthracis	Firmicutes
WP_036086548.1	4E-49	Leuconostoc pseudomesenteroides	Firmicutes
WP_025775188.1	5E-49	Moorella thermoacetica	Firmicutes
WP_016612337.1	6E-49	Enterococcus faecium	Firmicutes
EOO34434.1	6E-49	Bacillus cereus VDM019	Firmicutes
WP_013102215.1	1E-48	Leuconostoc kimchii	Firmicutes
WP_037283223.1	2E-48	Ruminococcus flavefaciens	Firmicutes
WP_002610711.1	3E-48	[Clostridium] innocuum	Firmicutes
WP_016526647.1	4E-48	Lactobacillus plantarum	Firmicutes
WP_022221722.1	4E-48	Firmicutes bacterium CAG:170	Firmicutes
WP_038670462.1	4E-48	Ruminococcus bicirculans	Firmicutes
WP_002349448.1	5E-48	Enterococcus faecium	Firmicutes
CDM14960.1	7E-48	Staphylococcus epidermidis PM221	Firmicutes
WP_014063579.1	8E-48	Thermoanaerobacter wiegelii	Firmicutes
CCO06330.1	9E-48	Ruminococcus bicirculans	Firmicutes
WP_028519085.1	9E-48	Ruminococcus flavefaciens	Firmicutes
WP_012110918.1	1E-47	Staphylococcus epidermidis	Firmicutes
WP_004913360.1	1E-47	Leuconostoc pseudomesenteroides	Firmicutes
WP_014757783.1	1E-47	Thermoanaerobacterium	Firmicutes
CCZ25005.1	2E-47	Coprobacillus sp. CAG:235	Firmicutes
WP_016626028.1	2E-47	Enterococcus faecalis	Firmicutes
WP_022638691.1	3E-47	Lactobacillus plantarum	Firmicutes
EJR98013.1	3E-47	Bacillus cereus VDM034	Firmicutes
WP_032089691.1	3E-47	bacterium LF-3	Undefined
WP_033125305.1	6E-47	Eubacterium sp. ER2	Firmicutes
WP_010829318.1	6E-47	Enterococcus faecalis	Firmicutes
ERJ43267.1	6E-47	Lactobacillus jensenii MD IIE-70(2)	Firmicutes
WP_010825638.1	6E-47	Enterococcus faecalis	Firmicutes
EFK80315.1	8E-47	Lactobacillus salivarius ACS-116-V-Col5a	Firmicutes
WP_003870381.1	1E-46	Thermoanaerobacter ethanolicus	Firmicutes
WP_009271167.1	2E-46	Erysipelotrichaceae bacterium 3_1_53	Firmicutes
WP_033799789.1	2E-46	Bacillus pseudomycoides	Firmicutes
WP_016627349.1	2E-46	Enterococcus faecalis	Firmicutes
WP_004915878.1	3E-46	Leuconostoc pseudomesenteroides	Firmicutes
WP_033799746.1	5E-46	Bacillus pseudomycoides	Firmicutes
WP_013275253.1	5E-46	Thermosediminibacter oceani	Firmicutes
WP_009609904.1	6E-46	Caldanaerobacter subterraneus	Firmicutes
WP_004899709.1	8E-46	Leuconostoc citreum	Firmicutes

WP_008727415.1	1E-45	Erysipelotrichaceae bacterium 2_2_44A	Firmicutes
WP_009490450.1	2E-45	Catellicoccus marimammalium	Firmicutes
WP_039107363.1	2E-45	Lactobacillus brevis	Firmicutes
WP_005727288.1	2E-45	Lactobacillus crispatus	Firmicutes
WP_021882777.1	2E-45	Ruminococcus sp. CAG:108	Firmicutes
WP_016526766.1	2E-45	Lactobacillus plantarum	Firmicutes
WP_002611774.1	2E-45	[Clostridium] innocuum	Firmicutes
WP_008789393.1	2E-45	Coprobacillus	Firmicutes
WP_035450183.1	3E-45	Lactobacillus crispatus	Firmicutes
WP_021355074.1	3E-45	Lactobacillus crispatus	Firmicutes
WP_005728983.1	4E-45	Lactobacillus crispatus	Firmicutes
WP_035163812.1	9E-45	Lactobacillus crispatus	Firmicutes
WP_020992884.1	9E-45	Lactobacillus crispatus	Firmicutes
WP_001832849.1	2E-44	Staphylococcus	Firmicutes
WP_013486320.1	3E-44	Ethanoligenens harbinense	Firmicutes
EEU20064.1	4E-44	Lactobacillus crispatus 125-2-CHN	Firmicutes
WP_028119414.1	4E-44	Facklamia sourekii	Firmicutes
EEX29575.1	7E-44	Lactobacillus crispatus MV-3A-US	Firmicutes
WP_024526869.1	1E-43	Lactobacillus brevis	Firmicutes
WP_011679807.1	1E-43	Leuconostoc mesenteroides	Firmicutes
KFL92914.1	2E-43	Lactobacillus crispatus SJ-3C-US	Firmicutes
ERK56596.1	2E-43	Oscillibacter sp. KLE 1745	Firmicutes
WP_035437706.1	3E-43	Lactobacillus fermentum	Firmicutes
WP_012695426.1	3E-43	Lactobacillus brevis	Firmicutes
WP_037282594.1	4E-43	Ruminococcus flavefaciens	Firmicutes
WP_033716994.1	7E-43	Bacillus cereus	Firmicutes
WP_009260026.1	1E-42	Lachnospiraceae bacterium 7_1_58FAA	Firmicutes
WP_022254068.1	1E-42	Firmicutes bacterium CAG:466	Firmicutes
AIR11519.1	1E-42	Lactobacillus salivarius	Firmicutes
WP_031907727.1	2E-42	Staphylococcus aureus	Firmicutes
EJY26930.1	2E-42	Enterococcus faecium 515	Firmicutes
WP_002502794.1	4E-42	Staphylococcus epidermidis	Firmicutes
WP_005727995.1	5E-42	Lactobacillus crispatus	Firmicutes
WP_002567435.1	5E-42	Lachnoclostridium	Firmicutes
WP_021966105.1	7E-42	Clostridium sp. CAG:149	Firmicutes
WP_008729120.1	9E-42	unclassified Erysipelotrichaceae (miscellaneous)	Firmicutes
WP_025705838.1	9E-42	Paenibacillus graminis	Firmicutes
WP_013499820.1	1E-41	Ruminococcus albus	Firmicutes
WP_026281184.1	1E-41	Paenibacillus massiliensis	Firmicutes
WP_025544413.1	1E-41	Oscillospiraceae bacterium VE202-24	Firmicutes
WP_034982192.1	2E-41	Lactobacillus salivarius	Firmicutes
WP_019123868.1	2E-41	Brevibacillus massiliensis	Firmicutes
WP_035301254.1	2E-41	Clostridium sp. ATCC BAA-442	Firmicutes
WP_021998148.1	3E-41	Ruminococcus sp. CAG:724	Firmicutes
WP_003699147.1	3E-41	Lactobacillus salivarius	Firmicutes
WP_036624164.1	5E-41	Paenibacillus macerans	Firmicutes
WP_022363987.1	6E-41	Clostridium sp. CAG:505	Firmicutes
WP_003708454.1	8E-41	Lactobacillus salivarius	Firmicutes
WP_004564444.1	8E-41	Lactobacillus salivarius	Firmicutes
WP_006354115.1	8E-41	[Clostridium] methylpentosum	Firmicutes
WP_000646815.1	1E-40	Bacillus cereus	Firmicutes
WP_006499474.1	2E-40	Lactobacillus mucosae	Firmicutes
WP_036602327.1	3E-40	Paenibacillus sophorae	Firmicutes
WP_024858116.1	3E-40	Ruminococcus albus	Firmicutes
WP_021729426.1	4E-40	Bacillus thuringiensis	Firmicutes

KID43098.1	4E-40	Lactobacillus brevis	Firmicutes
AIQ31451.1	5E-40	Paenibacillus sp. FSL P4-0081	Firmicutes
WP_017262375.1	5E-40	Lactobacillus rossiae	Firmicutes
EEL96047.1	6E-40	Bacillus mycoides DSM 2048	Firmicutes
WP_012008615.1	6E-40	Bacillus cereus group	Firmicutes
WP_025845227.1	7E-40	Paenibacillus ehimensis	Firmicutes
WP_039293300.1	7E-40	Paenibacillus sp. IHB B 3415	Firmicutes
EOP29673.1	9E-40	Bacillus cereus VD131	Firmicutes
WP_000646816.1	9E-40	Bacillus thuringiensis	Firmicutes
WP_003497096.1	1E-39	[Clostridium] symbiosum	Firmicutes
WP_035149403.1	3E-39	Lactobacillus salivarius	Firmicutes
EEM25643.1	3E-39	Bacillus thuringiensis Bt407	Firmicutes
AIQ13805.1	7E-39	Paenibacillus durus	Firmicutes
WP_004902803.1	3E-38	Leuconostoc citreum	Firmicutes
WP_033162705.1	7E-38	Sharpea azabuensis	Firmicutes
WP_024982822.1	8E-38	Brevibacillus borstelensis	Firmicutes
EJQ71226.1	1E-37	Bacillus cereus HuA4-10	Firmicutes
WP_028308371.1	2E-37	Clostridiales	Firmicutes
WP_016094009.1	2E-37	Bacillus cereus	Firmicutes
WP_002311868.1	2E-37	Enterococcus faecium	Firmicutes
WP_026826949.1	3E-37	Exiguobacterium sibiricum	Firmicutes
WP_009613734.1	3E-37	Desulfosporosinus sp. OT	Firmicutes
WP_010724523.1	3E-37	Enterococcus faecium	Firmicutes
KGJ51040.1	3E-37	[Clostridium] innocuum	Firmicutes
EOG03077.1	4E-37	Enterococcus faecium EnGen0170	Firmicutes
WP_029465370.1	5E-37	[Clostridium] hathewayi	Firmicutes
WP_036935414.1	1E-36	Pseudobacteroides cellulosolvens	Firmicutes
WP_010249462.1	1E-36	Acetivibrio cellulolyticus	Firmicutes
WP_003529350.1	2E-36	[Clostridium] leptum	Firmicutes
WP_011377372.1	3E-36	Enterococcus faecium	Firmicutes
AGN24556.1	3E-36	Erysipelothrix rhusiopathiae SY1027	Firmicutes
ELB16493.1	4E-36	Enterococcus faecium EnGen0025	Firmicutes
WP_020843008.1	6E-36	Lactobacillus reuteri	Firmicutes
WP_022420296.1	1E-35	Eubacterium dolichum CAG:375	Firmicutes
WP_028077616.1	2E-35	Solobacterium moorei	Firmicutes
WP_002351068.1	3E-35	Enterococcus faecium	Firmicutes
WP_033799479.1	3E-35	Bacillus pseudomycoides	Firmicutes
WP_033799774.1	3E-35	Bacillus pseudomycoides	Firmicutes
WP_005552659.1	5E-35	Paenibacillus alvei	Firmicutes
EHL12995.1	2E-34	Oribacterium parvum ACB1	Firmicutes
WP_011679815.1	7E-34	Leuconostoc mesenteroides	Firmicutes
GAK31595.1	7E-34	Weissella oryzae SG25	Firmicutes
CCC57136.1	7E-34	Weissella thailandensis fsh4-2	Firmicutes
WP_022221558.1	2E-33	Firmicutes bacterium CAG:170	Firmicutes
EEM13717.1	3E-33	Bacillus pseudomycoides DSM 12442	Firmicutes
EEM14088.1	4E-33	Bacillus pseudomycoides DSM 12442	Firmicutes
WP_038672375.1	1E-32	Ruminococcus bicirculans	Firmicutes
WP_014573607.1	1E-32	Lactobacillus salivarius	Firmicutes
WP_034864513.1	1E-32	[Clostridium] saccharogumia	Firmicutes
WP_017464865.1	2E-32	Staphylococcus epidermidis	Firmicutes
EJE21046.1	3E-32	Staphylococcus epidermidis NIHLM001	Firmicutes
WP_012477521.1	3E-32	Enterococcus faecium	Firmicutes
WP_034838882.1	3E-32	[Clostridium] cellulosi	Firmicutes
WP_002456271.1	4E-32	Staphylococcus	Firmicutes
WP_006771469.1	5E-32	[Clostridium] hathewayi	Firmicutes

WP_003654735.1	8E-32	Lactobacillus gasseri	Firmicutes
WP_015518753.1	1E-31	[Eubacterium] siraeum	Firmicutes
WP_021893319.1	1E-31	Clostridium bolteae CAG:59	Firmicutes
WP_028078749.1	2E-31	Solobacterium moorei	Firmicutes
ERI68515.1	2E-31	Clostridium sp. ATCC BAA-442	Firmicutes
KGF56411.1	3E-31	Clostridium orbiscindens 1_3_50AFAA	Firmicutes
WP_022271328.1	6E-31	Eubacterium siraeum CAG:80	Firmicutes
WP_021722612.1	8E-31	Lactococcus lactis	Firmicutes
WP_002611168.1	8E-31	[Clostridium] innocuum	Firmicutes
WP_035315839.1	2E-30	Brochothrix campestris	Firmicutes
WP_032607178.1	2E-30	Staphylococcus epidermidis	Firmicutes
WP_015567253.1	2E-30	[Eubacterium] siraeum	Firmicutes
WP_002144411.1	2E-30	Bacillus cereus group	Firmicutes
ENZ54723.1	2E-30	[Clostridium] bolteae 90A5	Firmicutes
EOP29672.1	2E-30	Bacillus cereus VD131	Firmicutes
WP_002502740.1	4E-30	Staphylococcus epidermidis	Firmicutes
WP_008791096.1	4E-30	Coprobacillus sp. 8_2_54BFAA	Firmicutes
WP_009009677.1	5E-30	Coprobacillus sp. D7	Firmicutes
WP_009327684.1	6E-30	Bacillus sp. 7_6_55CFAA_CT2	Firmicutes
WP_013274149.1	8E-30	[Clostridium] saccharolyticum	Firmicutes
WP_022288320.1	1E-29	Ruminococcus sp. CAG:57	Firmicutes
WP_021631180.1	2E-29	Clostridium sp. ATCC BAA-442	Firmicutes
WP_010691957.1	2E-29	Fructobacillus fructosus	Firmicutes
WP_002404191.1	3E-29	Enterococcus faecalis	Firmicutes
EJD78016.1	4E-29	Staphylococcus epidermidis NIHLM088	Firmicutes
WP_016104015.1	5E-29	Bacillus cereus	Firmicutes
WP_021951307.1	5E-29	Clostridium sp. CAG:678	Firmicutes
WP_032797867.1	7E-29	Lactobacillus paracasei	Firmicutes
WP_038672523.1	8E-29	Ruminococcus bicirculans	Firmicutes
WP_032797865.1	9E-29	Lactobacillus paracasei	Firmicutes
WP_002350204.1	1E-28	Enterococcus faecium	Firmicutes
WP_022434046.1	1E-28	Mycoplasma sp. CAG:472	Tenericutes
WP_002319908.1	1E-28	Enterococcus faecium	Firmicutes
WP_002485445.1	2E-28	Staphylococcus epidermidis	Firmicutes
WP_020368244.1	3E-28	Staphylococcus epidermidis	Firmicutes
EHM90319.1	5E-28	Coprobacillus sp. 3_3_56FAA	Firmicutes
EOH55681.1	2E-27	Enterococcus faecium EnGen0265	Firmicutes
EOP20053.1	2E-27	Bacillus cereus VD131	Firmicutes
WP_006526678.1	2E-27	Solobacterium moorei	Firmicutes
WP_007048755.1	4E-27	Subdoligranulum variabile	Firmicutes
WP_028078765.1	8E-27	Solobacterium moorei	Firmicutes
WP_021630851.1	1E-26	Clostridium sp. ATCC BAA-442	Firmicutes
EPC25263.1	2E-26	Lactobacillus paracasei subsp. paracasei Lpp22	Firmicutes
WP_009587655.1	3E-26	Clostridium sp. HGF2	Firmicutes
ELB58074.1	3E-26	Enterococcus faecium EnGen0052	Firmicutes
WP_025483103.1	4E-26	Clostridiales bacterium VE202-28	Firmicutes
WP_021630562.1	8E-26	Clostridium sp. ATCC BAA-442	Firmicutes
WP_020814081.1	2E-25	[Clostridium] papyrosolvens	Firmicutes
WP_015926503.1	5E-25	[Clostridium] cellulolyticum	Firmicutes
WP_022512780.1	1E-24	Clostridium clostridioforme CAG:511	Firmicutes
WP_024833866.1	1E-24	[Clostridium] josui	Firmicutes
WP_014312989.1	2E-24	Clostridium sp. BNL1100	Firmicutes
WP_011679978.1	4E-24	Leuconostoc mesenteroides	Firmicutes
WP_012096084.1	4E-24	Staphylococcus epidermidis	Firmicutes
EES57920.1	4E-24	Staphylococcus epidermidis BCM-HMP0060	Firmicutes

WP_002350132.1	4E-24	Enterococcus faecium	Firmicutes
WP_036596736.1	5E-24	Oribacterium parvum	Firmicutes
ERK59502.1	6E-24	Oscillibacter sp. KLE 1728	Firmicutes
WP_031392515.1	8E-24	Clostridium sp. KNHs209	Firmicutes
WP_021349865.1	1E-23	Lactobacillus fermentum	Firmicutes
WP_039293203.1	1E-23	Paenibacillus sp. IHB B 3415	Firmicutes
WP_009299812.1	2E-23	Coprobacillus sp. 3_3_56FAA	Firmicutes
WP_029744597.1	2E-23	Enterococcus faecium	Firmicutes
WP_009538200.1	2E-23	Oribacterium parvum	Firmicutes
ELA81368.1	2E-23	Enterococcus faecium EnGen0016	Firmicutes
EJY31312.1	2E-23	Enterococcus faecium 511	Firmicutes
WP_002351067.1	3E-23	Enterococcus faecium	Firmicutes
EOP20054.1	3E-23	Bacillus cereus VD131	Firmicutes
WP_002305028.1	4E-23	Enterococcus faecium	Firmicutes
WP_008792808.1	5E-23	Coprobacillus sp. 8_2_54BFAA	Firmicutes
WP_029482395.1	7E-23	Clostridiales bacterium VE202-18	Firmicutes
WP_004625365.1	7E-23	[Clostridium] termitidis	Firmicutes
WP_007494767.1	9E-23	Flavonifractor plautii	Firmicutes
EPC25261.1	1E-22	Lactobacillus paracasei subsp. paracasei Lpp22	Firmicutes
WP_016112043.1	2E-22	Bacillus cereus	Firmicutes
WP_022789871.1	3E-22	Faecalicoccus pleomorphus	Firmicutes
WP_029376513.1	5E-22	Staphylococcus epidermidis	Firmicutes
EPC47597.1	3E-21	Lactobacillus paracasei subsp. paracasei Lpp123	Firmicutes
EPC49498.1	4E-21	Lactobacillus paracasei subsp. paracasei Lpp123	Firmicutes
WP_023568877.1	1E-20	Staphylococcus epidermidis	Firmicutes
WP_039833923.1	6E-20	Paenibacillus sonchi	Firmicutes
WP_021121838.1	2E-19	[Clostridium] sordellii	Firmicutes
WP_034565784.1	2E-19	Clostridiales bacterium VE202-03	Firmicutes
WP_021894497.1	1E-18	Clostridium bolteae CAG:59	Firmicutes
EFD08427.1	2E-18	Enterococcus faecium D344SRF	Firmicutes
EJY25571.1	1E-17	Enterococcus faecium 514	Firmicutes
WP_032799415.1	1E-17	Streptococcus sobrinus	Firmicutes
EJE26573.1	3E-17	Staphylococcus epidermidis NIHLM001	Firmicutes
WP_021751938.1	4E-17	Oscillibacter sp. KLE 1745	Firmicutes
WP_021121827.1	7E-17	[Clostridium] sordellii	Firmicutes
WP_016370176.1	1E-16	Lactobacillus paracasei	Firmicutes
WP_002351396.1	1E-16	Enterococcus faecium	Firmicutes
WP_006875585.1	2E-16	Anaerotruncus colihominis	Firmicutes
WP_003605669.1	2E-16	Lactobacillus casei	Firmicutes
WP_031239643.1	2E-16	Streptococcus iniae	Firmicutes
WP_021121834.1	2E-16	[Clostridium] sordellii	Firmicutes
EPC22773.1	2E-16	Lactobacillus paracasei subsp. paracasei Lpp226	Firmicutes
WP_010688001.1	3E-16	Leuconostoc gelidum	Firmicutes
WP_039141255.1	3E-16	Lactobacillus rhamnosus	Firmicutes
WP_016370207.1	5E-16	Lactobacillus paracasei	Firmicutes
EEL96048.1	6E-16	Bacillus mycoides DSM 2048	Firmicutes
WP_002350930.1	7E-16	Enterococcus faecium	Firmicutes
WP_016112044.1	1E-15	Bacillus cereus	Firmicutes
