# Supplementary material for: Discovery of a new family of relaxases in Firmicutes bacteria
Source: PLoS Genet. 2017 Feb 16;13(2):e1006586. doi: 10.1371/journal.pgen.1006586 (PMC5313138; doi:10.1371/journal.pgen.1006586)
Supplement: S2 Table — (DOCX) [file pgen.1006586.s008.docx]

**Supplemental information**

| **Supplemental Table S2.** Occurrence (%) of MOB_L_ signatures in members of other MOB families (%) | | | | | | |
| --- | --- | --- | --- | --- | --- | --- |
| **MOB_L_ signature** | **MOB_Q_** | **MOB_V_** | **MOB_P_** | **MOB_F_** | **MOB_C_** | **MOB_H_** |
| **1** | 32 | 82 | 70 | - | - | - |
| **2** | - | - | 2 | - | - | - |
| **3** | - | - | - | - | - | - |
| **4** | - | - | 2 | - | - | - |
| **5** | - | - | 5 | - | - | - |
| **6** | - | - | 1 | 2 | - | - |
| **7** | - | - | 2 | - | - | - |
| **8** | - | - | 2 | - | - | - |
| **9** | 2 | - | - | 4 | - | - |
| **10** | - | - | 1 | - | - | - |
