# Supplementary material for: Discovery of a new family of relaxases in Firmicutes bacteria
Source: PLoS Genet. 2017 Feb 16;13(2):e1006586. doi: 10.1371/journal.pgen.1006586 (PMC5313138; doi:10.1371/journal.pgen.1006586)
Supplement: S3 Table — (DOCX) [file pgen.1006586.s009.docx]

**Supplemental information**

| **Supplemental Table S3.** Occurrence (%) of the signatures of the MOB_Q_, MOB_V_, MOB_P_, MOB_F_, MOB_C_ and MOB_H_ in members of the MOB_L_ family | | | | | | |
| --- | --- | --- | --- | --- | --- | --- |
| **signature** | **MOB_Q_** | **MOB_V_** | **MOB_P_** | **MOB_F_** | **MOB_C_** | **MOB_H_** |
| **1** | 0.3 | 4 | 85 | - | - | - |
| **2** | - | - | 2 | - | - | - |
| **3** | - | - | - | - |  | - |
| **4** | - | - | 10 | - |  |  |
| **5** | - | - | 7 | - |  |  |
| **6** | 1 | - | - | - |  |  |
| **7** | - | 2 | - | - |  |  |
| **8** | - | - | - | - |  |  |
| **9** | - | - | - | - |  |  |
| **10** | - | - | - | - |  |  |
