# Supplementary material for: Discovery of a new family of relaxases in Firmicutes bacteria
Source: PLoS Genet. 2017 Feb 16;13(2):e1006586. doi: 10.1371/journal.pgen.1006586 (PMC5313138; doi:10.1371/journal.pgen.1006586)
Supplement: S4 Table — (DOCX) [file pgen.1006586.s010.docx]

**Supplemental information**

| **Supplemental TableS4. Strains used** | | |
| --- | --- | --- |
| **Strains** | **genotype or description** | **Source or Reference** |
| *E. coli* |  |  |
| XL1-Blue | *end*A1 *gyr*A96(nal^R^) *thi*-1 *rec*A1 *rel*A1 *lac* *gln*V44 F'[ ::Tn10 *pro*AB^+^ *lac*I^q^ Δ(*lac*Z)M15] *hsd*R17 (r_K_^-^ m_K_^+^) | [1] |
| BL21(DE3) | F^–^ *ompT* *gal* *dcm* *lon* *hsdS_B_*(*r_B_*^–^*m_B_*^–^) λ(DE3 [*lacI* *lacUV5*-*T7 gene 1* *ind1* *sam7* *nin5*]) [*malB*^+^]_K-12_(λ^S^) | laboratory stock |
| AZ37 | XL1-Blue strain harboring pET28b+ derivative pAND83 (containing *rel_LS20_His_(6)_*) | This work |
| AZ42 | BL21(DE3) strain harboring pET28b+ derivative pAND83 (containing *rel_LS20_His_(6)_*) | This work |
| AZ38 | XL1-Blue strain harboring pET28b+ derivative pAND84 (containing *N-rel_LS20_His_(6)_*) | This work |
| AZ43 | BL21(DE3) strain harboring pET28b+ derivative pAND84 (containing *N-rel_LS20_His_(6)_*) | This work |
| BF28 | XL1-Blue strain harboring pET28b+ derivative pCG108 (containing *rel_LS20_Y26F-His_(6)_*) | This work |
| BF29 | BL21(DE3) strain harboring pET28b+ derivative pCG1084 (containing *rel_LS20_Y26F-His_(6)_*) | This work |
| *B. subtilis* |  |  |
| 168 (1A700) | *trpC2* | BGSC* |
| PKS11 | 168 harboring pLS20cat | [2] |
| GR81 | *trpC2*, pUCTA2501 (Em) | This work |
| GR104 | *trpC2*, pUCTA2501 (Em), pLS20cat (Cm) | This work |
| GR114 | *trpC2,* pGR10A (Em),pLS20cat (Cm) | This work |
| GR115 | *trpC2,*pGR12A (Em),pLS20cat (Cm) | This work |
| GR121 | *trpC2,* pGR10B (Em), pLS20cat (Cm) | This work |
| GR122 | *trpC2,* pGR12B (Em), pLS20cat (Cm) | This work |
| GR124 | *trpC2,* pGR8A (Em), pLS20cat (Cm) | This work |
| GR126 | *trpC2*, *amyE*::P*_spank_*-56-58 (Spec) | This work |
| GR137 | *trpC2,* pGR16A (Em), pLS20cat (Cm) | This work |
| GR138 | *trpC2,* pGR16B (Em), pLS20cat (Cm) | This work |
| GR139 | *trpC2,* pGR22A (Em), pLS20cat (Cm) | This work |
| GR140 | *trpC2,* pGR20B (Em), pLS20cat (Cm) | This work |
| GR149 | *trpC2,* pLS20Δ56-58 (Km, Cm) | This work |
| GR150 | *trpC2*, *amyE*::P*_spank_*-56-58 (Spec), pLS20Δ56-58 (Km, Cm) | This work |
| GR183 | *trpC2,* pGR8B (Em), pLS20cat (Cm) | This work |
| GR184 | *trpC2,* pGR20A (Em), pLS20cat (Cm) | This work |
| GR185 | *trpC2,* pGR22B (Em), pLS20cat (Cm) | This work |
| GR206 | *trpC2,* *amy*E::P_spank_-*56-57* (*spec*), pLS20Δ56-58 (Km, Cm) | This work |
| *, BGSC:  *Bacillus* Genetic Stock Center, Department of Bioch*em*istry, The Ohio State University, Columbus, OH, USA. (<http://www.bgsc.org/> | | |

**References**

1. Bullock WO, Fernandez JM, Short JM (1987) XL1-blue: a high efficiency plasmid transforming *recA* *Escherichia coli* strain with Beta-galactosidase selection. Biotechniques 5: 376-379.

2. Singh PK, Ramachandran G, Duran-Alcalde L, Alonso C, Wu LJ, Meijer WJ (2012) Inhibition of *Bacillus subtilis* natural competence by a native, conjugative plasmid-encoded *comK* repressor protein. Environ Microbiol 14: 2812-2825. 10.1111/j.1462-2920.2012.02819.x [doi].
