# Supplementary material for: Discovery of a new family of relaxases in Firmicutes bacteria
Source: PLoS Genet. 2017 Feb 16;13(2):e1006586. doi: 10.1371/journal.pgen.1006586 (PMC5313138; doi:10.1371/journal.pgen.1006586)
Supplement: S5 Table — (DOCX) [file pgen.1006586.s011.docx]

| **Supplemental Table S5. Plasmids used** | | |
| --- | --- | --- |
| **Plasmid** | **Description** | **Reference or source** |
| pLS20cat | Native plasmid pLS20 labelled with Cm resistance cassette in the unique *Sal*I site | [1] |
| pDR110 | *B. subtilis amyE* integration vector containing IPTG-inducible P*_spank_* promoter (Spec) | D. Rudner* |
| pBEST501 | *E. coli* vector containing neomycin/kanamycin resistance marker in multiple cloning site | [2] |
| pLS20Δ56-58 | pLS20cat derivative in which genes *56-58* are replaced by kanamycin resistance gene of pBEST501 | This work |
| pUCTA2501 | pUC19 derivative containing replication functions of low copy number *B. subtilis* plasmid pTA1015 and erythromycin resistance gene of pE194 | This work |
| pET28b+ | Vector for expressing His-tagged labeled proteins in *E. coli* | Novagene, Madison, WI, USA |
| pGR8A | pLS20cat Fragment 1 cloned in vector pUCTA2501 to define *oriT_LS20_* (orientation A) | This work |
| pGR8B | pLS20cat Fragment 1 cloned in vector pUCTA2501 to define *oriT_LS20_* (orientation B) | This work |
| pGR10A | pLS20cat Fragment 2 cloned in vector pUCTA2501 to define *oriT_LS20_* (orientation A) | This work |
| pGR10B | pLS20cat Fragment 2 cloned in vector pUCTA2501 to define *oriT_LS20_* (orientation B) | This work |
| pGR12A | pLS20cat Fragment 3 cloned in vector pUCTA2501 to define *oriT_LS20_* (orientation A) | This work |
| pGR12B | pLS20cat Fragment 3 cloned in vector pUCTA2501 to define *oriT_LS20_* (orientation B) | This work |
| pGR16A | pLS20cat Fragment 6 cloned in vector pUCTA2501 to define *oriT_LS20_*  (orientation A) | This work |
| pGR16B | pLS20cat Fragment 6 cloned in vector pUCTA2501 to define oriT_LS20_ (orientation B) | This work |
| pGR20A | pLS20cat Fragment 4 cloned in vector pUCTA2501 to define *oriT_LS20_* (orientation A) | This work |
| pGR20B | pLS20cat Fragment 4 cloned in vector pUCTA2501 to define *oriT_LS20_* (orientation B) | This work |
| pGR22A | pLS20cat Fragment 5 cloned in vector pUCTA2501 to define *oriT_LS20_* (orientation A) | This work |
| pGR22B | pLS20cat Fragment 5 cloned in vector pUCTA2501 to define *oriT_LS20_* (orientation B) | This work |
| pGR27 | pDR110 derivative containing pLS20cat genes *56*, *57* and *58* behind the IPTG-inducible P*_spank_* promoter | This work |
| pGR52 | pDR110 derivative containing pLS20cat genes *56* and *57* behind the IPTG-inducible P*_spank_* promoter | This work |
| pAND83 | pET28b+ expression vector containing pLS20cat gene 58 (*rel_LS20_*) cloned in *Nco*I-*Sal*I sites to generate *rel_LS20_His_(6)_* fusion gene | This work |
| pAND84 | pET28b+ expression vector containing first 232 codons of pLS20cat gene 58 (*N-rel_LS20_*) cloned in *Nco*I-*Sal*I sites to generate *N-rel_LS20_His_(6)_* fusion gene | This work |
| pCG108 | pET28b+ expression vector containing pLS20cat gene 58 (*rel_LS20_*) harbouring mutation Y26F cloned in *Nco*I-*Sal*I sites to generate *rel_LS20_Y26FHis_(6)_* fusion gene | This work |
| *: D. Rudner, Department of Microbiology and Immunobiology, Harvard Medical School, 77 Avenue Louis Pasteur, Boston, MA 02115, USA | | |

**References**

1. Itaya M, Sakaya N, Matsunaga S, Fujita K, Kaneko S (2006) Conjugational transfer kinetics of pLS20 between *Bacillus subtilis* in liquid medium. Biosci Biotechnol Biochem 70: 740-742. JST.JSTAGE/bbb/70.740 [pii].

2. Itaya M, Kondo K, Tanaka T (1989) A neomycin resistance gene cassette selectable in a single copy state in the *Bacillus subtilis* chromosome. Nucleic Acids Res 17: 4410.
