# Supplementary material for: Discovery of a new family of relaxases in Firmicutes bacteria
Source: PLoS Genet. 2017 Feb 16;13(2):e1006586. doi: 10.1371/journal.pgen.1006586 (PMC5313138; doi:10.1371/journal.pgen.1006586)
Supplement: S6 Table — (DOCX) [file pgen.1006586.s012.docx]

| **Supplemental Table S6. Oligonucleotides used** | | |
| --- | --- | --- |
| Name | Sequence (5’-3’) | Purpose |
| ori-up | ccccggatcCAGGCCCGGGGCTTTACGTCAA | checking transconjugants or transformants for the presence of pLS20cat, in combination with ori-dn |
| ori-dn | ttttggatccGGGAATAACAGTATACGTTAGTG | checking transconjugants or transformants for the presence of pLS20cat, in combination with ori-up |
| oGR20 | tttttctagaTTCTGATAATCTCGCTTTCATTTCATCGTG | Cloning *oriT_LS20_* Fragment 1 in plasmid pUCTA2501 in combination with oGR21 |
| oGR21 | tttttctagaCCGAAAAAGTGAAAATAAAATTTA | Cloning  *oriT_LS20_* Fragment 1 in plasmid pUCTA2501 in combination with oGR20 |
| oGR22 | tttttctagaCTAAATAATGGTTGAACATAAATGT | Cloning *oriT_LS20_* Fragment 2 in plasmid pUCTA2501 with oGR20 |
| oGR23 | tttttctagaGTACCAGATTTATTGCTGAATGCA | Cloning  *oriT_LS20_* Fragment 3 in plasmid pUCTA2501 in combination with oGR21 |
| oGR27 | **ttttgcatgcTTATTTTTCACCTTTCTGCATTTCC** | Clonging pLS20cat genes *56* and *57* in pDR110 in combination with oGR133 |
| oGR28 | tttttctagaAAAGAGCAATCTCGTCATCGAAGAC | Cloning *oriT_LS20_* Fragment 5 in plasmid pUCTA2501in combination with oGR20; cloning *oriT_LS20_* Fragment 6 in plasmid pUCTA2501in combination with oGR29, determination *nic* site in *oriT_LS20_* |
| oGR29 | tttttctagaTTGTTAACGCTCCTTTTCATCGATTT | Cloning *oriT_LS20_* Fragment 4 in plasmid pUCTA2501 in combination with oGR21; cloning Fragment 6 in plasmid pUCTA2501in combination with oGR28; determination *nic* site in *oriT_LS20_* |
| oGR43 | ttttgctagcCAAAGTAATGTGCAGAAATCGATGA | Cloning genes *56, 57* and *58* in vector pDR110 in combination with oGR60 |
| oGR56 | GAAATCAAAGTGACATTTTAAAGGGGATCT | Deletion of genes *56-58* on pLS20cat |
| oGR57 | ttttgtcgacTTGAAAGACCTTTGATGTTGAGATCCGGCA | Deletion of genes *56-58* on pLS20cat |
| oGR58 | ttttggatccAGTATGAAATGGAACAGAGCCGGTAGGCAA | Deletion of genes *56-58* on pLS20cat |
| oGR59 | TGCCGTATGTTTGATACAGTTCTAAATATT | Deletion of genes *56-58* on pLS20cat |
| oGR60 | ttttgcatgcTCCTTTAATTTCAGAATTGCCTACC | Cloning pLS20cat genes *56-58* in vector pDR110 in combination with with oGR43 |
| oGR133 | ttttactagtCAAAGTAATGTGCAGAAATCGATGA | Cloning pLS20cat genes *56* and *57* in vector pDR110 in combination with oGR27 |
| oWM001 | ggggccatggATTCTCCTGGCGTTGTATTAGTCTCT | Cloning *rel_LS20_* in pET28b+ in combination with oWM002 |
| oWM002 | aaaagtcgacCCGGCTCTGTTCCATTTCATACTGC | Cloning *rel_LS20_* in pET28b+ in combination with oWM001 |
| oWM003 | aaaagtcgacCTGAAATTTAAGTCCTTTAGGAGC | Cloning *N-rel_LS20_* in pET28b+ in combination with oWM001 |
| oWM001A | GGCAAATCAACAAAATTTTCGAAGTATGTCAATT**T**TATCAATCGTGATGAAGCCGTCCG | Generating Y26F mutation in relLS20 in combination with oWM002 |
| oWM001B | CTCCTGGCGTTGTATTAGTCTCTAAATATGTGTCGGGCAAATCAACAAAATTTTCGAAGTATGTC | Extending 5´ region of the mutation generating Y26F in combination with 0WM002 |
| oJAH1a | CAGATTCTCTACAAGATATTCCTCCAAA | Generation 600 bp fragment F1 in combination with oJAH1b; to study bending *oriT_LS20_* region |
| oJAH1b | GTTTTCCATACAGAAATTTAGTCTTCGATG | Generation 600 bp fragment F1 in combination with oJAH1a; to study bending *oriT_LS20_* region |
| oJAH2a | TGATTTTTATGTGTTGTTTGTAAGAGAT | Generation 600 bp fragment F2 in combination with oJAH2b; to study bending *oriT_LS20_* region |
| oJAH2b | GCTGAATGCACATAAAAAAAACATTTATG | Generation 600 bp fragment F2 in combination with oJAH2a; to study bending *oriT_LS20_* region. |
| oJAH3a | TCGAGCAAAAAGATATATTCCTCATCTGC | Generation 600 bp fragment F3 in combination with oJAH3b; to study bending *oriT_LS20_* region |
| oJAH3b | GAAGGAGGGGGTGGAAAAGGAAAGAGCATAAGGGG | Generation 600 bp fragment F3 in combination with oJAH3a; to study bending *oriT_LS20_* region. |
| oJAH4a | AGATCAAAAATAATAAAAAATTGATTCA | Generation 600 bp fragment F4 in combination with oJAH4b; to study bending *oriT_LS20_* region |
| oJAH4b | CATCGATTTCTGCACATTACTTTGCAACG | Generation 600 bp fragment F4 in combination with oJAH4a; to study bending *oriT_LS20_* region. |
| oJAH5a | AGAAGATAAGGAATACCAGATCGGTAAAG | Generation 600 bp fragment F5 in combination with oJAH5b; to study bending *oriT_LS20_* region |
| oJAH5b | TGAGAAAGTCCTGCCTTCCTCGCTT | Generation 600 bp fragment F5 in combination with oJAH5a; to study bending *oriT_LS20_* region. |
| oJAH6a | ACGAGCAAAGAAATGCTAGGTGAACTTG | Generation 600 bp fragment F6 in combination with oJAH6b; to study bending *oriT_LS20_* region. |
| oJAH6b | GAATGCTACAGATTTAATGACTTCTCCT | Generation 600 bp fragment F6 in combination with oJAH6a; to study bending *oriT_LS20_* region |
| oJAH7a | CAGTTATTTTTCGTGTGCATAAAATAAAG | Generation 600 bp fragment F7 in combination with oJAH7b; to study bending *oriT_LS20_* region. |
| oJAH7b | TGCTGCTTTAAAAAGAAATAAGGACAGCGTCCT | Generation 600 bp fragment F7 in combination with oJAH7a; to study bending *oriT_LS20_* region |
| oJAH8a | CAATAAATCTGGTACCACGAAAAAACAAACCGC | Generation 600 bp fragment F8 in combination with oJAH8b; to study bending *oriT_LS20_* region. |
| oJAH8b | ACAGCACAAAATGATCTTTGCAGCTCATT | Generation 600 bp fragment F8 in combination with oJAH8a; to study bending *oriT_LS20_* region |
| oWM001 | ggggccatggATTCTCCTGGCGTTGTATTAGTCTCT | Cloning *rel_LS20_* in pET28b+ in combination with oWM002 |
| oWM002 | aaaagtcgacCCGGCTCTGTTCCATTTCATACTGC | Cloning *rel_LS20_* in pET28b+ in combination with oWM001 |
|  |  |  |
| 5´- overhang sequences are indicated in lower case and restriction sites are underlined | | |
